# Tuning CO_2_ Absorption in Hydrophobic Protic Ionic Liquids via Temperature and Structure

**DOI:** 10.3390/molecules30244674

**Published:** 2025-12-05

**Authors:** Nurin Athirah Mohd Mazlan, Madelyn Wen Qian Teoh, Asyraf Hanim Ab Rahim, Gani Purwiandono, Normawati M. Yunus

**Affiliations:** 1 Department of Applied Science, Universiti Teknologi PETRONAS, Seri Iskandar 32610, Perak, Malaysia; 2 Nanotechnology and Catalysis Research Centre (NANOCAT), Universiti Malaya, 50603 Kuala Lumpur, Malaysia; 3 Centre for Research in Ionic Liquid (CORIL), Institute of Sustainable Energy and Resources (ISER), Universiti Teknologi PETRONAS, Seri Iskandar 32610, Perak, Malaysia; 4 Department of Chemistry, Universitas Islam Indonesia, Sleman 55584, Indonesia

**Keywords:** protic ionic liquids, CO_2_ absorption, free volume, hydrophobic, recyclability

## Abstract

Conventional amine-based solvents such as monoethanolamine (MEA) and diethanolamine (DEA) are widely used for CO_2_ removal from natural gas but this technology suffers from drawbacks including high regeneration energy, solvent degradation, and corrosion issues. To overcome these limitations, this study investigates the use of newly synthesized hydrophobic protic ionic liquids (HPILs) composed of ammonium cations coupled with the bis(trifluoromethane)sulfonylimide ([Tf_2_N]^−^) anion for CO_2_ absorption using the pressure-drop method. The results show that CO_2_ solubility increases with pressure but decreases with temperature. Among the studied ionic liquids (ILs), [BEHA][Tf_2_N] exhibits the highest CO_2_ capacity at 298.15 K within the pressure range of 1–20 bar, which is consistent with its free volume (*V_f_*) value. Furthermore, a comparison study indicates that all ILs demonstrate superior CO_2_ selectivity over methane (CH_4_) at 298.15 K. The recyclability study shows that [BEHA][Tf_2_N] maintains its structural integrity over two CO_2_ absorption cycles at 20 bar across all tested temperatures.

## 1. Introduction

Carbon dioxide (CO_2_) is a greenhouse gas that significantly contributes to global environmental issues such as global warming and climate change [1,2]. With the mitigation of CO_2_ emissions becoming a key focus in the energy sector, natural gas has been recognized as a cleaner alternative energy source for electricity generation, heating and industrial applications. However, natural gas not only contains methane (CH_4_) as its main component but also acidic gases including CO_2_ and hydrogen sulphide (H_2_S) [3]. The presence of CO_2_ in natural gas reduces its heating value and promotes corrosion in pipelines, making its removal essential for both economic and safety reasons [4]. The separation of CO_2_ from methane (CH_4_) is a crucial step in producing natural gas that is rich in calorific value [5]. The removal of CO_2_ using amine scrubbing technology, which utilizes solvent such as monoethanolamine (MEA) and diethanolamine (DEA), remains the most commercially favourable approach in large-scale facilities due to its high CO_2_ selectivity and affordable and fast reaction kinetics [6].

Nevertheless, this technology faces several drawbacks, including the requirement for high regeneration energy, solvent degradation, and equipment corrosion [7,8,9]. These drawbacks have encouraged the development of materials such as ionic liquids (ILs), deep eutectic solvents (DES), soft organic materials [10] and metal–organic frameworks (MOFs). Several new processes are also being developed, including membrane separation and cryogenic processing [11,12,13,14]. However, membrane separation still suffers from limited selectivity and permeability and is very sensitive to impurities. On the other hand, while cryogenic technologies can produce high-purity CO_2_, they require high energy consumption and complex refrigeration systems, thus making them less economical for low-concentration CO_2_ streams. Among the various gas capture techniques, absorption and adsorption are the most widely investigated. According to Soo et al., absorption refers to the dissolution of CO_2_ into a liquid medium, which may occur via either chemisorption or physisorption. In contrast, adsorption involves the attachment of CO_2_ molecules to the surface of a solid material [15]. The absorption of CO_2_ using ILs has attracted significant attention due to its negligible vapour pressure, high thermal stability, and tunable chemical structures [7].

Over the past few decades, numerous ILs have been synthesized and studied for CO_2_ absorption. These include room-temperature ionic liquids (RTILs) and task-specific ionic liquids (TSILs), both of which have shown strong potential for CO_2_ capture [16,17]. However, their large-scale application is limited by their high production costs and the use of hazardous reagents during synthesis [8]. Protic ILs (PILs) offer a more accessible alternative, as they can be synthesized through a simple acid–base neutralization reaction without the use of organic solvents [18]. Some PILs have demonstrated CO_2_ uptake comparable to that of conventional ILs, although their relatively low thermal stability remains a challenge for industrial applications. Consequently, the synthesis of hydrophobic protic ionic liquids (HPILs) which incorporate non-polar groups to improve stability, reduce water absorption, and enhance CO_2_ selectivity, has been proposed. While there are limited studies on the utilization of HPILs in gas capture, Huang and co-workers reported the use of hydrophobic ILs for H_2_S removal, recording a high absorption capacity of 0.546 mol/mol at 1 bar [19]. This further highlights the potential of HPILs for acid gas separation.

**Figure 1:**
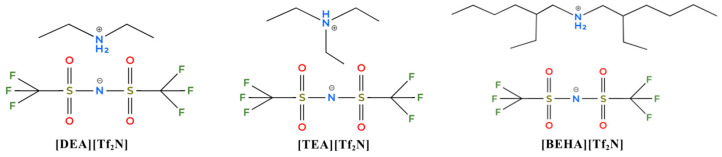
Chemical structures of the synthesized HPILs ([DEA][Tf_2_N], [TEA][Tf_2_N], and [BEHA][Tf_2_N]).

Thus, this work reports the synthesis and characterization of three new hydrophobic PILs (HPILs) with different cation structures, namely diethylammonium bis-(trifluoromethane)sulfonylimide [DEA][Tf_2_N], triethylammonium bis-(trifluoromethane)sulfonylimide [TEA][Tf_2_N], and bis(2-ethylhexyl)ammonium bis-(trifluoromethane)sulfonylimide [BEHA][Tf_2_N], as shown in Figure 1. The purity of the HPILs was confirmed by structural analysis and their thermal properties were tested. This paper also provides a detailed study on the thermophysical properties of HPILs, including molar volume (*V_m_*), free volume (*V_f_*), and lattice energy (*U_latt_*). CO_2_ solubility was measured at pressures from 1 to 20 bars and at temperatures of 298.15 K, 313.15 K, and 333.15 K. Recyclability was also studied to evaluate the potential of these HPILs for CO_2_ capture. In addition, CO_2_ absorption was compared with methane (CH_4_) absorption at 298.15 K under similar pressure conditions.

## 2. Results and Discussion

### 2.1. Structural Confirmation

The structures of the synthesized PILs and HPILs were confirmed using ^1^H NMR, ^13^C NMR, and FTIR spectroscopy. The obtained data were consistent with the expected molecular structures. All NMR chemical shifts and spectra are provided in the Supplementary Materials (Figures S1–S12).

#### FTIR Analysis

**Figure 2:**
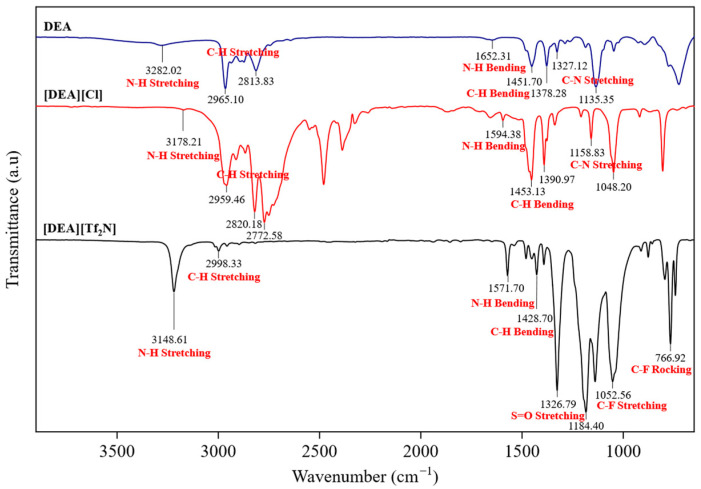
FTIR spectra of DEA, [DEA][Cl], and [DEA][Tf_2_N].

The FTIR spectra of DEA, [DEA][Cl], and [DEA][Tf_2_N] confirm the successful formation of the protic ILs, as shown in Figure 2. All other FTIR spectra are provided in the Supplementary Materials, Figures S13 and S14. For PILs, N-H stretching bands were detected in the range 3178–3404 cm^−1^, together with bending vibrations between 1590 and 1650 cm^−1^ [20]. These shifts, when compared with the parent amines, indicate proton transfer and the formation of ammonium species. C-H stretching bands appeared between 2960 and 2850 cm^−1^, with corresponding bending modes at 1450–1380 cm^−1^, confirming that the alkyl groups remained unchanged after protonation. C-N stretching was observed between 1150 and 1035 cm^−1^ [21]. For HPILs, additional peaks were present, including a strong absorption at 1340 cm^−1^ assigned to S=O stretching [20,21], several intense bands between 1180 and 1050 cm^−1^ from C-F vibrations, and a band near 740 cm^−1^ corresponding to C-F rocking. The presence of these characteristic S=O and C-F vibrations in the HPILs further supports the incorporation of the [Tf_2_N]^−^ anion and confirms the successful synthesis of the HPILs.

### 2.2. Thermal Stability of PILs and HPILs

The thermal stability of the synthesized PILs and HPILs was evaluated using thermogravimetric analysis (TGA). The onset temperature (*T_onset_*) marks the beginning of decomposition, while the peak temperature (*T_max_*) corresponds to the maximum decomposition rate. The results are presented in Table 1.

The decomposition temperature of the PILs and HPILs in this work was evaluated based on *T_onset_*, which represents the point at which thermal degradation begins. For the PILs, [TEA][Cl] exhibited a slightly higher *T_onset_*, indicating marginally greater thermal stability compared with [DEA][Cl] and [BEHA][Cl]. The small differences in their *T_onset_* values suggest that the chloride anion primarily governs the thermal behaviour, rather than the cation. In contrast, the HPILs displayed significantly higher *T_onset_* values, ranging from 614.15 to 672.15 K, compared with 478.15 to 480.15 K for the PILs. Among the HPILs, [TEA][Tf_2_N] was the most thermally stable, followed by [DEA][Tf_2_N] and [BEHA][Tf_2_N]. Their enhanced stability is attributed to the bulky, charge-delocalized [Tf_2_N]^−^ anion, which reduces localized charge density and delays the onset of decomposition relative to chloride [22]. Similar improvements in thermal resistance associated with bulky or delocalized anions have been reported for ammonium-based PILs [7,23].

Despite being bulkier, [BEHA][Tf_2_N] is less thermally stable than [TEA][Tf_2_N]. The long, branched 2-ethylhexyl chains of [BEHA]^+^ cation increase *V_f_* and weaken cation–anion interactions, leading to earlier decomposition. In contrast, the more compact [TEA]^+^ cation forms stronger interactions with [Tf_2_N]^−^, resulting in enhanced stability. This behaviour is consistent with previous reports showing that longer alkyl chains reduce IL stability by weakening ion–ion interactions [23].

### 2.3. Heat Capacity

**Figure 3:**
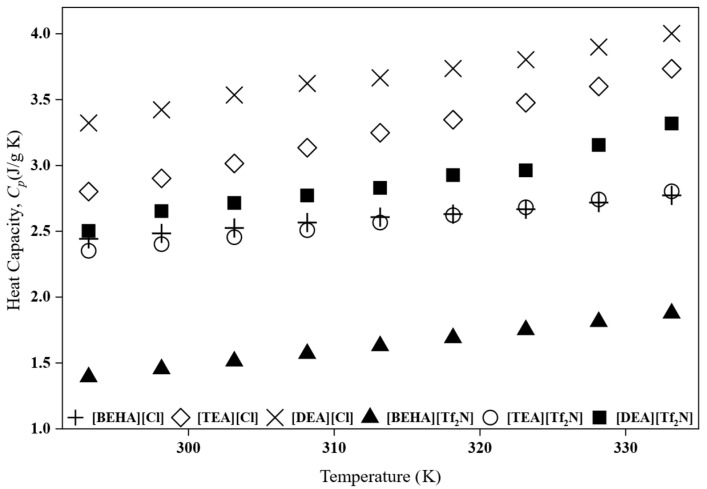
Heat capacity, *C_p_*, of PILs and HPILs at 293.15 K–333.15 K.

The heat capacity of PILs and HPILs is an important property because it affects how much energy is needed during the regeneration step in separation processes [7]. However, there are still limited data available for PILs and HPILs. In this study, the specific heat capacity (*C_p_*) of the synthesized PILs and HPILs was measured using DSC, and the results are presented in Figure 3. Besides, the values of heat capacity can be found in the Supplementary Materials (Table S1). All samples exhibited an increase in *C_p_* with temperature, consistent with trends reported in previous studies for both protic and aprotic ILs [7,24,25].

Importantly, the *C_p_* values of all PILs studied were consistently lower than those of the conventional 30 wt% monoethanolamine (MEA) aqueous solution, which typically ranges from 3.9 to 4.3 J g^−1^ K^−1^ over the same temperature interval [7]. This lower heat capacity suggests reduced sensible heat requirements during regeneration, thereby improving the overall energy efficiency of CO_2_ absorption processes [26].

### 2.4. Density

**Figure 4:**
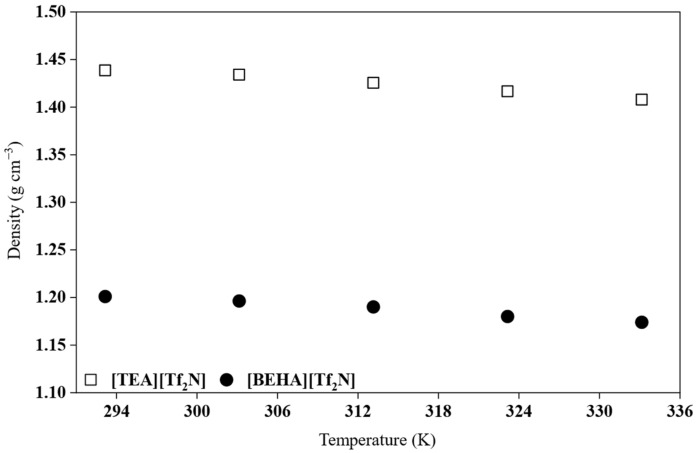
Experimental density variation in [TEA][Tf_2_N] and [BEHA][Tf_2_N] as a function of temperature.

Density is known to be temperature-dependent, and the density of ILs typically decreases with increasing temperature [27,28]. As the temperature increases, ions gain kinetic energy, leading to greater intermolecular separation. This expansion increases the free volume within the liquid and results in lower density values. In this study, the densities of the HPILs that exist in the liquid state, namely [BEHA][Tf_2_N] and [TEA][Tf_2_N], were measured in triplicate, and the results are summarized in Table S2 of the Supplementary Materials. Figure 4 illustrates the average density values of [TEA][Tf_2_N] and [BEHA][Tf_2_N] within the temperature range of 293.15 to 333.15 K, whereas Table 2 lists the densities of [DEA][Tf_2_N], [TEA][Tf_2_N], and [BEHA][Tf_2_N] at 298.15 K.

The density of the investigated HPILs, as shown in Table 2, follows the order [DEA][Tf_2_N] > [TEA][Tf_2_N] > [BEHA][Tf_2_N]. This trend reflects the influence of cation structure on packing efficiency [28,29]. The highest density of [DEA][Tf_2_N] is due to the small and compact diethylammonium cation, which allows for closer interaction with the [Tf_2_N]^−^ anion. The presence of two N-H groups also promotes hydrogen bonding, leading to stronger attraction between ions and a lower free volume. The [TEA]^+^ cation, with three ethyl substituents and only one N–H group, exhibits reduced hydrogen-bonding capacity and steric hindrance, resulting in intermediate density values. In contrast, the bulky [BEHA]^+^ cation, bearing two long branched alkyl chains, introduces significant free volume and weaker ionic interactions, leading to the lowest density. This observation is consistent with the findings of Zailani and co-workers, who reported that smaller cations enhance local packing and increase density, whereas longer alkyl chains introduce steric hindrance and reduce density values [9]. Similar trends have also been found in other ILs, such as pyridinium and imidazolium systems, where increasing the alkyl chain length decreases density due to higher molecular asymmetry and free volume [8,30].

The density variation with temperature was correlated using the linear relationship shown in Equation (1):

(1) $$\rho =D_{1}T+D_{0}$$

where ρ represents the density (g·cm^−3^), *T* is the absolute temperature (K), and $D_{1}$ and $D_{0}$ are the regression coefficients obtained by the least-squares method. The goodness of fit was assessed by calculating the standard deviation (*SD*) using Equation (2), where $x_{exp}$ and $x_{calc}$ are the experimental and calculated values, respectively, and *N* is the number of experimental data points. The regression coefficients, together with the corresponding *SD* values, are presented in Table 3.

(2) $$SD=\sqrt{\frac{\sum_{i}^{nDAT}{\left(x_{exp}-x_{calc}\right)}^{2}}{N}}$$

The thermal expansion coefficient (α) was obtained using Equation (3), and the calculated values are summarized in Table 4. The α represents the relative change in density with temperature and indicates how much the ionic liquid expands when heated. In this study, all three HPILs exhibited α values in the order of 10^−4^ K^−1^, which is in good agreement with previously reported ranges for ILs [7].

(3) $$\alpha =\left(\frac{1}{\rho }\right)\times \left(\frac{\partial \rho }{\partial T}\right)=-(\frac{D_{1}}{D_{0}+D_{1}T})$$

Within the studied range of 293–333 K, α values remained nearly constant, confirming that thermal expansion in HPILs is weakly temperature-dependent under moderate heating, as also observed for other ILs in the literature [7,9]. In addition, the experimental density data were used to calculate the molar volume ($V_{m}$) and the molecular volume (*V*) according to the equations below:

(4) $$V_{m}=\frac{M}{\rho }$$

(5) $$V=\frac{M}{\rho \times N_{A}}$$

The $V_{m}$ was obtained from the ratio of molar mass (M) to density, while the *V* was calculated by dividing $V_{m}$ by Avogadro’s number. According to Glasser’s theory, these parameters were further employed to estimate the standard molar entropy (*S°*) and the lattice energy (*U_latt_*) using the following equations:

(6) S°=1246.5×V+29.5

(7) $$U_{latt}=1981.2\times {\left(\frac{\rho }{M}\right)}^{\frac{1}{3}}+103.8$$

Based on Table 4, the results reveal that the $V_{m}$ and *V* increase in the order of [TEA][Tf_2_N] < [BEHA][Tf_2_N], in agreement with increasing cation size and *V_f_*. Correspondingly, the Glasser-derived thermodynamic properties indicate that [TEA][Tf_2_N] has the highest lattice energy and lowest entropy, reflecting its compact ionic structure and crystalline state. In contrast, [BEHA][Tf_2_N] exhibits the lowest lattice energy and highest entropy, consistent with its bulky, flexible cation and reduced packing efficiency [31].

### 2.5. Refractive Index

**Figure 5:**
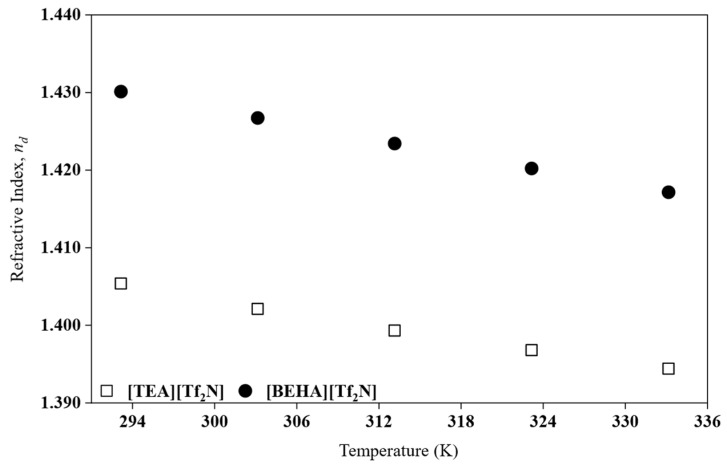
The *n_d_* plot for HPILs at a temperature range of 293.15 K to 333.15 K.

In this work, refractive index measurements were carried out for HPILs that exist as liquid at room temperature, namely [TEA][Tf_2_N] and [BEHA][Tf_2_N]. The analysis was conducted in the temperature range of 293.15 to 333.15 K. The experimental data are presented in Figure 5. The refractive index values for both HPILs are summarized in Table S3 of the Supplementary Materials. The refractive index values, *n_d_* of the synthesized HPILs were fitted to a linear equation using the least-squares method, based on Equation (8), and the standard deviation (*SD*) was calculated accordingly. In Equation (8), $R_{1}$ and $R_{0}$ represent the estimated fitting parameters. The corresponding parameters and *SD* values are listed in Table 5, enabling the estimation of $n_{d}$ at other temperatures.

(8) $$n_{d}=R_{1}T+R_{0}$$

As shown in Figure 5, the *n_d_* decreases with increasing temperature. This behaviour is consistent with previous studies on PILs [7]. Between the two liquids, [BEHA][Tf_2_N] exhibits a higher *n_d_* than [TEA][Tf_2_N] even though its density is lower. This apparent contradiction can be explained by considering that the *n_d_* is influenced not only by density but also by molar refraction ($R_{m}$), which is related to the polarizability of the ions [32]. From the *n_d_*, the $R_{m}$ or electronic polarizability was calculated by employing the Lorentz–Lorenz relation, as shown in Equation (9).

(9) $$R_{m}=\left(\frac{{n_{d}}^{2}-1}{{n_{d}}^{2}+2}\right)V_{m}$$

where $V_{m}$ is the molar volume obtained from density data. The calculated $R_{m}$ values of [BEHA][Tf_2_N] are higher than those of [TEA][Tf_2_N] based on Table 4, confirming that the bulkier BEHA cation has greater electronic polarizability. The $R_{m}$ values do not vary significantly with temperature, indicating that molar refraction is not strongly dependent on temperature. In addition, the obtained $R_{m}$ values can be further used to calculate the free volume ($V_{f}$) based on Equation (10).

(10) $$V_{f}=V_{m}-R_{m}$$

Based on Table 4, the results show that [BEHA][Tf_2_N] has a larger *V_f_* than [TEA][Tf_2_N], which is consistent with its lower density and reflects less efficient packing of the longer alkyl chains [29]. The combination of higher $R_{m}$ and larger *V_f_* explains why [BEHA][Tf_2_N] maintains a higher *n_d_* despite its lower density. Similar behaviour has been reported for both protic and aprotic ILs, where longer alkyl chains decrease density but enhance refractive index due to increased polarizability [23,33,34].

Although the *n_d_* of [DEA][Tf_2_N] was not experimentally determined in its present state, its smaller cation size suggests a lower $R_{m}$ compared to [TEA][Tf_2_N] and [BEHA][Tf_2_N]. Consequently, [DEA][Tf_2_N] would be expected to exhibit the lowest r *n_d_* of the series. This inference is consistent with the general relationship between cation size, $R_{m}$ and *n_d_* reported by Zailani et al. [23] for DEA-based PILs. However, *n_d_* data for PILs in the solid state for further comparison and analysis are still limited in the literature.

### 2.6. CO_2_ Absorption Analysis

**Figure 6:**
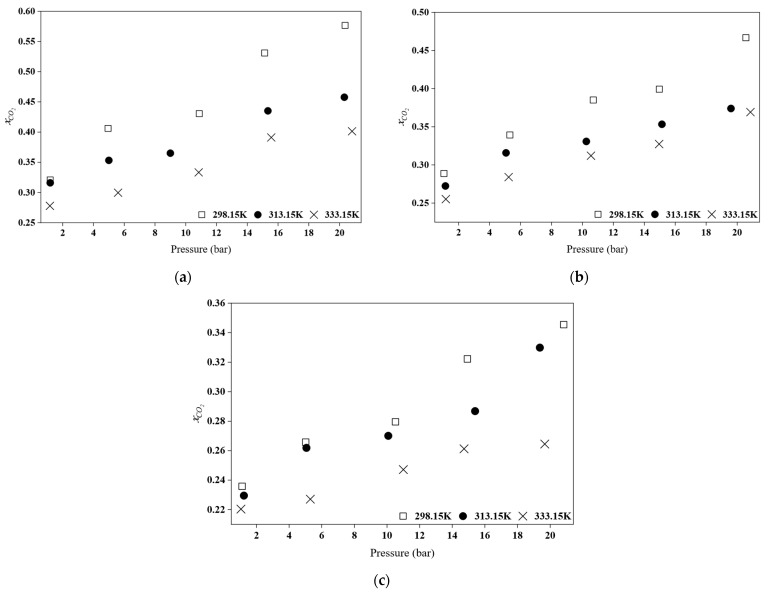
Comparison of CO_2_ absorption for (**a**) [BEHA][Tf_2_N], (**b**) [TEA][Tf_2_N], and (**c**) [DEA][Tf_2_N] at different temperatures.

**Figure 7:**
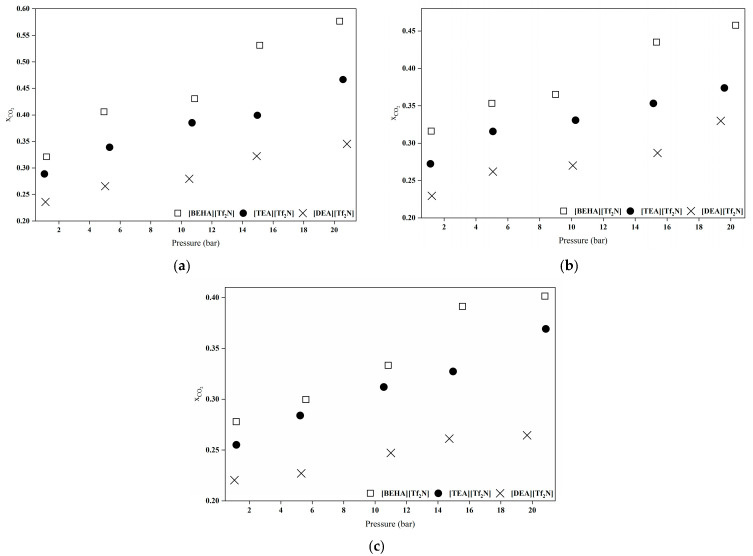
Comparison of CO_2_ absorption capacities for all HPILs at varying temperatures: (**a**) 298.15 K, (**b**) 313.15 K, and (**c**) 333.15 K.

In this study, the CO_2_ absorption of three newly synthesized HPILs, namely [DEA][Tf_2_N], [TEA][Tf_2_N], and [BEHA][Tf_2_N], was analyzed at different temperatures of 298.15 K, 313.15 K, and 333.15 K over a pressure range of 1 bar to 20 bar. The CO_2_ absorption data are presented in terms of mole fraction ($x_{{CO}_{2}}$) and the results are shown in Figure 6 and Figure 7. The Henry’s law constants (*K_H_*) for CO_2_ solubility in the HPILs were obtained by linear regression of Equation (16), and the corresponding values for all investigated temperatures are summarized in Table 6. The solubility characteristics of CO_2_ in the synthesized [Tf_2_N]-based ILs are discussed in the following sections.

#### 2.6.1. Effect of Pressure and Temperature

Based on Figure 6 and Figure 7, an increase in pressure leads to an increase in the mol fraction of CO_2_ absorption. This aligns with the Henry’s Law, which states that an increase in pressure results in a higher CO_2_ absorption capacity [7,35]. The solubility of a gas in a liquid is proportional to the pressure, indicating a physical process [8]. Furthermore, the effect of temperature on the solubility of CO_2_ in this HPILs was also studied at 298.15 K, 313.15 K, and 333.15 K, as illustrated in Figure 6. As shown by the data, an increase in temperature leads to a decrease in CO_2_ absorption capacity [36,37]. A similar trend was reported for pyridinium-based ILs studied by Yunus and co-workers [8]. It was observed that the effect of temperature is more pronounced at high pressure. This is due to the decreased gas solubility at higher temperatures as the increased kinetic energy allows gas molecules to escape more readily from the liquid phase [8,38].

The temperature derivative of the solubility is related to the either the partial molar enthalpy, $\Delta h_{2}$, or the partial molar entropy, $\Delta s_{2}$, of the gaseous solute in the liquid phase. The enthalpy and entropy change in solution provide information on the effect of temperature on solubility. Specifically, the enthalpy reflects the strength of interactions between the liquid and the dissolved gas, while the entropy indicates the degree of ordering in the liquid–gas mixture [8,39]. The values for $\Delta h_{2}$ and $\Delta s_{2}$ can be estimated from a linear fit data using Equations (11) and (12).

(11) $$\Delta h_{2}=-R{\left(\frac{\partial \ln x_{2}}{\partial \left(\frac{1}{T}\right)}\right)}_{P}=R{\left(\frac{\partial \ln K_{H}}{\partial \left(\frac{1}{T}\right)}\right)}_{P}$$

(12) $$\Delta s_{2}=R{\left(\frac{\partial \ln x_{2}}{\partial \ln T}\right)}_{P}=-R{\left(\frac{\partial \ln K_{H}}{\partial \ln T}\right)}_{P}$$

The $\Delta h_{2}$ and $\Delta s_{2}$ values for CO_2_ absorption in the studied HPILs are summarized in Table 7. All HPILs exhibit negative enthalpy and entropy values, indicating an exothermic process with increased molecular ordering upon absorption. The enthalpy values become more negative from [DEA][Tf_2_N] to [BEHA][Tf_2_N], indicating stronger interactions with CO_2_ as the cation size increases [8,37]. This trend may be attributed to the longer alkyl chains in [BEHA][Tf_2_N], which provide greater space and flexibility for CO_2_ incorporation. Although the enthalpy values in this work are more negative than those reported for conventional physical solvents such as Solexol (−15 to −20 kJ mol^−1^) [40], they remain within the typical range for ionic liquids undergoing physisorption. For example, Hu et al. reported enthalpy values between −3.801 and −4.729 kJ mol^−1^ for CO_2_ absorption [41], supporting the presence of moderately exothermic interactions in ionic liquid systems. The negative entropy values, ranging from −10.02 to −22.38 J mol^−1^ K^−1^, further indicate that CO_2_ becomes more ordered within the ionic liquids [8].

#### 2.6.2. Effect of Cation Size

As shown in Figure 7, the CO_2_ absorption capacity of the HPILs follows the order of [BEHA][Tf_2_N] > [TEA][Tf_2_N] > [DEA][Tf_2_N]. This trend is influenced by cation size and structure, which affect the free volume (*V_f_*) and CO_2_ solubility. A strong linear correlation was observed between CO_2_ solubility and *V_f_* [7]. The [BEHA]^+^ cation contains long branched alkyl chains and has the highest molar mass among the studied HPILs. This creates a more open and less densely packed ionic liquid structure, providing additional space for CO_2_ molecules to be accommodated compared to [TEA][Tf_2_N] and [DEA][Tf_2_N] [8,34]. According to Aki et al. [42] and Yunus et al. [8], this behaviour is primarily driven by entropic factors rather than enthalpic contributions. The observed decrease in density and the corresponding increase in entropy from [DEA][Tf_2_N] to [BEHA][Tf_2_N] confirm that the enhanced CO_2_ solubility arises from the greater structural flexibility and increased free space within the ionic liquid system.

### 2.7. Gas Solubility Comparison Between CO_2_ and CH_4_ in the HPILs

**Figure 8:**
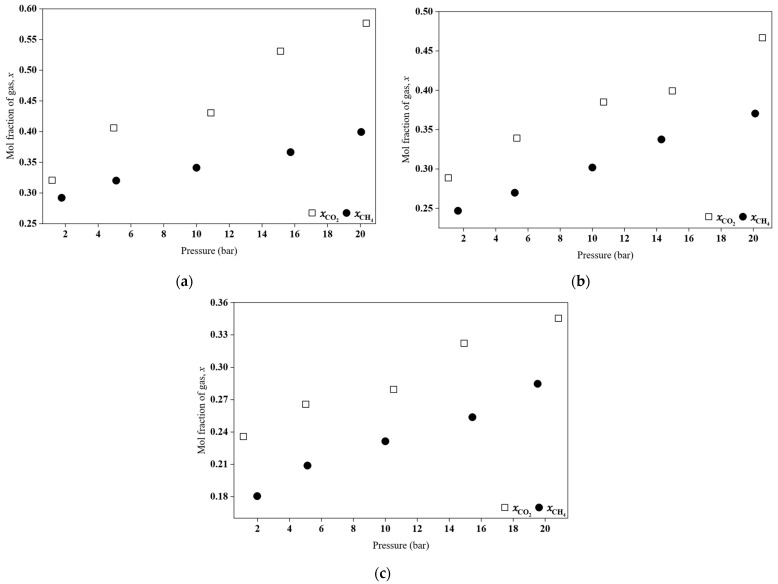
Comparison of CO_2_ and CH_4_ absorption behaviour for the synthesized HPILs at 298.15 K: (**a**) [BEHA][Tf_2_N], (**b**) [TEA][Tf_2_N], and (**c**) [DEA][Tf_2_N].

The solubility of CO_2_ and methane (CH_4_) in [BEHA][Tf_2_N], [TEA][Tf_2_N], and [DEA][Tf_2_N] at 298.15 K was determined, and the results are presented in Figure 8. As shown, all the HPILs absorbed significantly higher amounts of CO_2_ compared to CH_4_. The Henry’s law constants (*K_H_*) for both gases are summarized in Table 8. For all systems, the *K_H_* values of CO_2_ were lower than those of CH_4_, confirming that CO_2_ is more soluble in these HPILs.

The difference in solubility is attributed to the molecular characteristics of the gases and their interactions with the HPILs. CO_2_, which has a permanent quadrupole moment and moderate polarizability, interacts strongly with HPIL components, particularly the [Tf_2_N]^−^ anion, through Lewis acid–base and dipole–quadrupole interactions. These interactions enhance physical absorption and result in a lower Henry’s constant. In contrast, CH_4_ is nonpolar and has very low polarizability, interacting weakly with the HPIL through dispersion forces and exhibiting lower solubility. This behaviour, in which CO_2_ shows higher solubility than CH_4_ due to its affinity for charged and polar groups, has been widely reported in the literature [43,44,45].

### 2.8. Comparison with Other Reported Ionic Liquids

For comparison, few studies have reported the CO_2_ solubility in HPILs composed of ammonium cations paired with the bis(trifluoromethylsulfonyl)imide [Tf_2_N]^−^ anion. Thus, the present CO_2_ solubility data were compared with related ILs, including 1-hexyl-3-methylimidazolium bis(trifluoromethylsulfonyl)imide ([HMIM][Tf_2_N]) and 1-butylpyridinium bis(trifluoromethylsulfonyl)imide ([C_4_Py][Tf_2_N]). At 298.15 K, [BEHA][Tf_2_N] exhibited a CO_2_ mole fraction of 0.5766, higher than the 0.415 observed for [HMIM][Tf_2_N] under similar conditions [7]. This indicates a greater affinity for CO_2_, likely due to weaker cation–anion interactions in ammonium-based HPILs. A comparison of the *K_H_* values further confirms the higher CO_2_ solubility of ammonium-based PILs. For pyridinium-based ILs [C_4_Py][Tf_2_N], Yunus et al. [8] reported *K_H_* values of 40.7 bar at 313.15 K and 51.7 bar at 333.15 K. In contrast, the present [DEA][Tf_2_N] showed lower *K_H_* values of 39.29 and 44.09 bar at the same respective temperatures, suggesting that the shorter-alkyl ammonium cation enhances CO_2_ absorption more effectively than the aromatic pyridinium structure. The lower *K_H_* values of [DEA][Tf_2_N] are attributed to the flexible alkyl environment, which provides increased free volume and reduced ion-pairing strength, facilitating greater physical absorption of CO_2_.

Further comparison between identical cations paired with different anions supports the CO_2_-philic nature of [Tf_2_N]^−^. Yunus et al. [46] reported a CO_2_ mole fraction of 0.4860 at 298.15 K and 20 bar for bis(2-ethylhexyl)ammonium butyrate ([BEHA][BA]), which is lower than the 0.5766 mole fraction obtained by [BEHA][Tf_2_N] in this study. Moisture does not significantly influence CO_2_ uptake in ILs; for instance, Goodrich et al. showed that even 14 wt% water in [P_66614_][Met] reduced CO_2_ absorption to only 0.2 mol CO_2_ per mol IL [47]. Although [BEHA][Tf_2_N] contains slightly more water (0.48%) than [BEHA][BA] (0.15%), it still exhibits superior CO_2_ uptake, indicating that absorption is primarily governed by the anion rather than by moisture content. The large, weakly coordinating [Tf_2_N]^−^ anion, with its delocalized charge and highly fluorinated structure, enhances CO_2_-philicity through quadrupole–dipole interactions with CO_2_ molecules [48,49,50]. The combined effect of the flexible ammonium cation and the fluorinated [Tf_2_N]^−^ anion provides a synergistic improvement in CO_2_ absorption compared with previously reported ammonium, pyridinium, and imidazolium systems. Task-specific ILs containing amine functionalities can chemically capture CO_2_, but their strong solvent–solute interactions often require higher regeneration energy [8]. In contrast, the [Tf_2_N]-based HPILs synthesized in this work demonstrate excellent physical absorption performance, achieving a favourable balance between solubility and regenerability, as discussed in the following section. These findings highlight the potential of ammonium-based PILs as efficient and energy-favourable alternatives for CO_2_ capture under mild conditions.

### 2.9. Characterization of HPILs After CO_2_ Absorption

**Figure 9:**
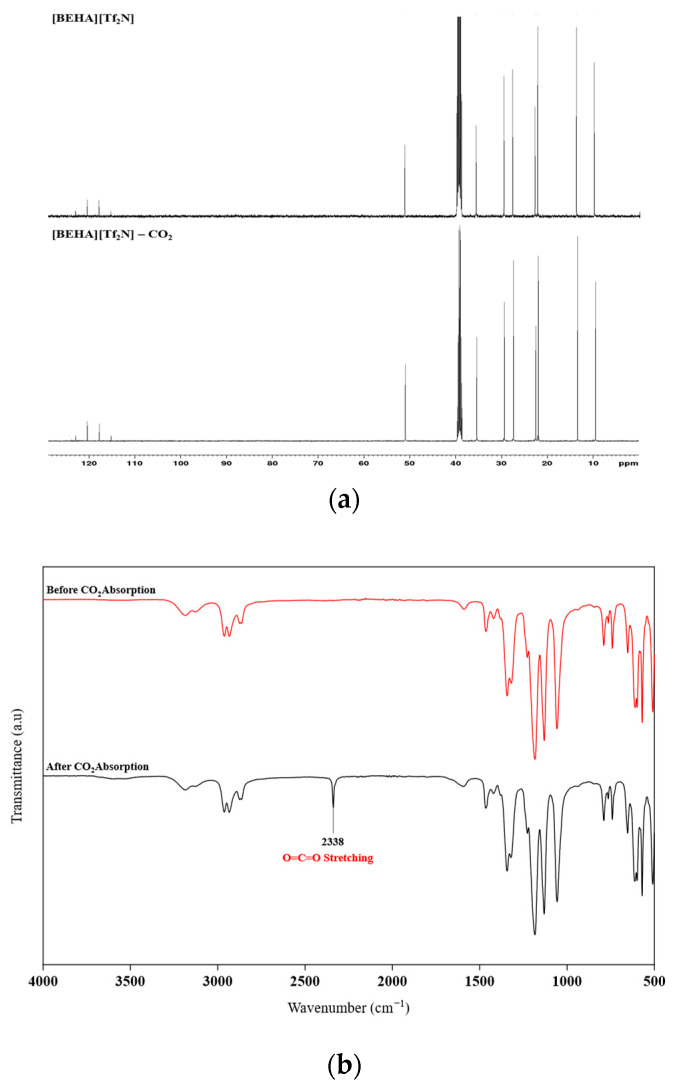
Spectroscopic characterization of [BEHA][Tf_2_N] before and after CO_2_ absorption: (**a**) stacked ^13^C NMR spectra and (**b**) FTIR spectra recorded using the ATR method in the range of 3900–500 cm^−1^.

In this work, FTIR and ^13^C NMR spectroscopy were also employed to examine the possible CO_2_ absorption mechanism of the HPILs. For [BEHA][Tf_2_N], the ^13^C NMR spectrum based on Figure 9a shows no new signals after CO_2_ absorption, suggesting no chemical interaction between CO_2_ and HPILs. FTIR spectra were recorded using the ATR method in the range of 3900–500 cm^−1^. The spectrum of [BEHA][Tf_2_N] is presented in Figure 9b, while those of [TEA][Tf_2_N] and [DEA][Tf_2_N] are provided in the Supplementary Materials (Figures S15 and S16). Before and after CO_2_ absorption, the spectra were nearly identical, except for the appearance of a new band at 2337–2339 cm^−1^, attributed to the O=C=O stretching vibration of CO_2_. No additional peaks were observed in the carbonyl region around 1700–1720 cm^−1^, where carbamate formation would typically appear in a chemisorption process. These results confirm that CO_2_ uptake by the three HPILs occurs through physisorption rather than chemisorption.

### 2.10. Recyclability Performance of [BEHA][Tf_2_N]

**Figure 10:**
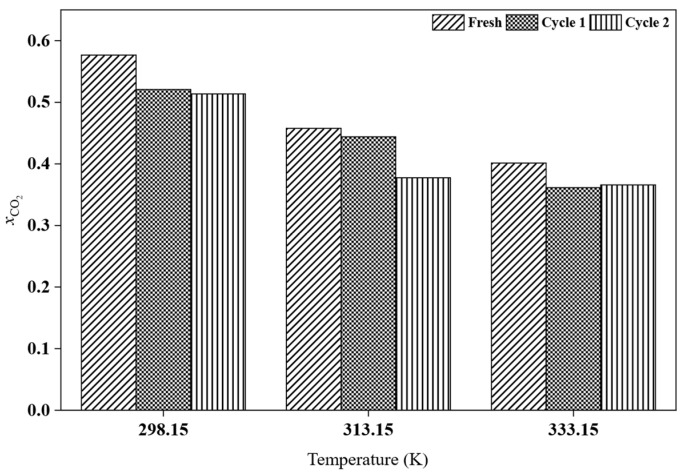
Recyclability of [BEHA][Tf_2_N] at 298.15 K, 313.15 K, and 333.15 K over two consecutive CO_2_ absorption–desorption cycles.

**Figure 11:**
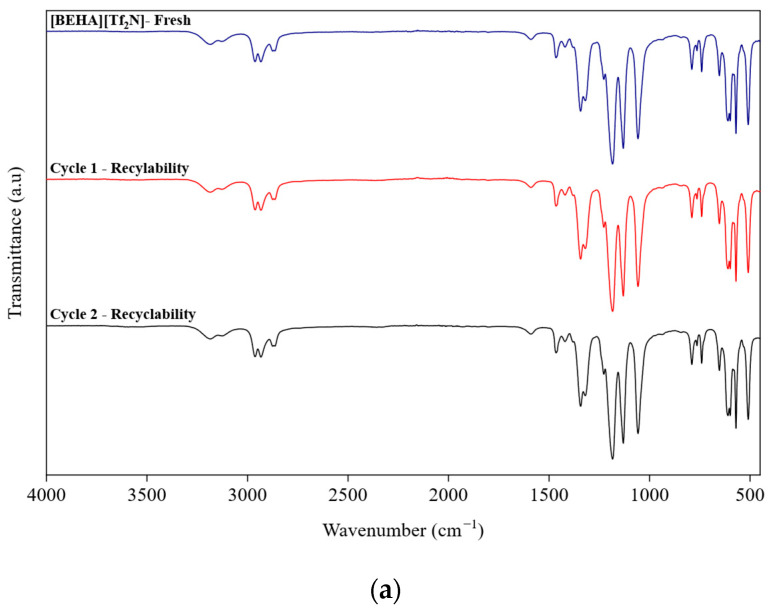

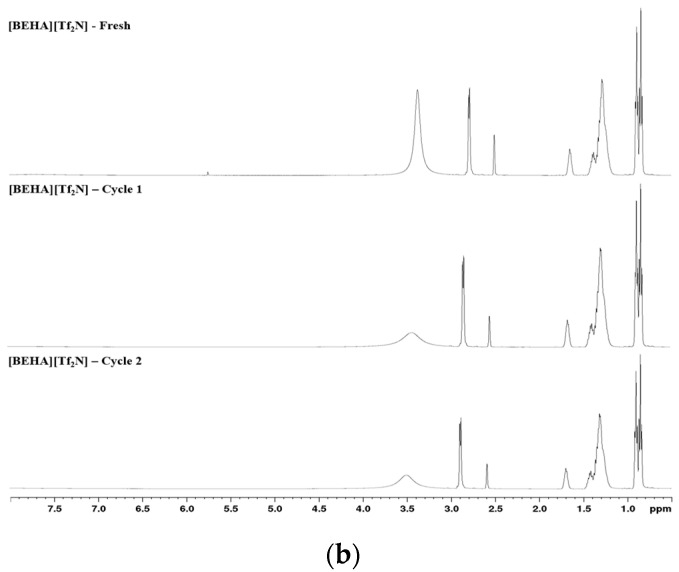
Spectroscopic characterization of [BEHA][Tf_2_N] after CO_2_ desorption: (**a**) FTIR spectra and (**b**) ^1^H NMR spectra for fresh, cycle 1, and cycle 2 samples at 298.15 K.

After CO_2_ absorption, the recyclability of the best-performing HPIL was further evaluated to assess its potential for reuse, considering that conventional amine scrubbing processes are costly and require advanced technologies. Among all the investigated HPILs, [BEHA][Tf_2_N] exhibited the highest CO_2_ solubility, at 20 bar. Therefore, recyclability tests for [BEHA][Tf_2_N] were conducted at 20 bar for two consecutive absorption–desorption cycles at 298.15 K, 313.15 K, and 333.15 K. After each cycle, the used [BEHA][Tf_2_N] was regenerated by heating at 333.15 K for 4 h to release the absorbed CO_2_ and then reused in the subsequent cycle. FTIR and ^1^H NMR analyses were also performed after CO_2_ desorption to evaluate the structural stability of [BEHA][Tf_2_N]. As shown in Figure 10, the recyclability plots show only slight variations in CO_2_ uptake between the two absorption–desorption cycles at all tested temperatures, indicating that [BEHA][Tf_2_N] has good stability and can be effectively reused for CO_2_ capture. Representative FTIR and ^1^H NMR spectra after CO_2_ desorption at 298.15 K are shown in Figure 11, while the corresponding spectra at 313.15 K and 333.15 K are provided in the Supplementary Materials (Figures S17–S20). The ^1^H NMR signals and FTIR peaks after two cycles remained essentially unchanged compared to the fresh sample, confirming that the molecular structure of [BEHA][Tf_2_N] was preserved throughout the recycling process.

## 3. Materials and Methods

### 3.1. Materials and Reagents

All chemicals of analytical grade were used without an additional purification process for the synthesis of HPILs. The CAS number, source, and chemical purity are as follows: diethylamine (109-89-7, Merck, Rahway, NJ, USA, 99%), triethylamine (121-44-8, Merck, Rahway, NJ, USA, 99%), bis(2-ethylhexyl)amine (106-20-7, Aldrich Chemistry, Saint Louis, MO, USA 99%), hydrochloric acid (7647-01-0, Fischer Scientific, Waltham, MA, USA 37%), lithium bis(trifluoromethylsulfonyl)imide (90076-65-6, Sigma Aldrich, St. Louis, MA, USA, 99%), dichloromethane (75-09-2, Merck, Rahway, NJ, USA, 99%).

### 3.2. Synthesis of Hydrophobic Protic Ionic Liquids (HPILs)

**Figure 12:**
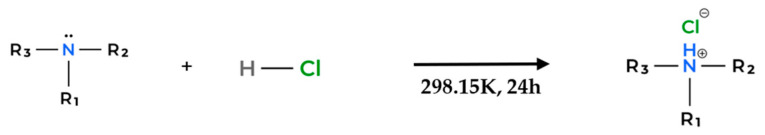
General synthesis pathway for first-step reaction to produce PIL.

**Figure 13:**
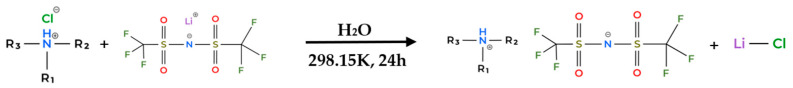
General synthesis pathway for second-step reaction to produce HPIL.

The HPILs in this study were synthesized through a two-step procedure, as illustrated in Figure 12 and Figure 13. PILs were synthesized before they were converted to HPILs. In the first step, an equimolar amount of hydrochloric acid was added dropwise into a 250 mL, three-neck, round-bottom flask containing the selected amines, which were triethylamine (TEA), diethylamine (DEA), or bis(2-ethylhexyl)amine (BEHA), while the flask was kept in an ice bath to control the temperature. The mixture was stirred at 250 rpm for 24 h at room temperature to ensure complete reaction. This step produced PILs, namely [DEA][Cl] and [TEA][Cl], as solids, and [BEHA][Cl] as a liquid. The products were dried using a rotary evaporator and stored in a vacuum cabinet until further use.

In the second step, the dried PILs from the first step was reacted with an equimolar amount of lithium bis(trifluoromethylsulfonyl)imide (LiTf_2_N) in 30 mL of deionized water. The mixtures were stirred at room temperature for 24 h for [TEA][Tf_2_N] and [DEA][Tf_2_N], and 48 h for [BEHA][Tf_2_N] due to its longer alkyl chain. After the reaction was completed, the IL phase was extracted using dichloromethane, and the solvent was removed by rotary evaporation. This step produced liquid products for all HPILs except [DEA][Tf_2_N], which remained as a solid.

### 3.3. Characterization

#### 3.3.1. Structural Analysis

The structures of the PILs and HPILs were confirmed using ^1^H and ^13^C Nuclear Magnetic Resonance (NMR) and Fourier Transform Infrared (FTIR) spectroscopy. For NMR analysis, approximately 5 mg of the dried ionic liquid was dissolved in 650 µL of deuterated solvent, transferred into an NMR tube, and analyzed at room temperature using a Bruker Avance III 500 MHz spectrometer Billerica, MA, USA. Chemical shifts (*δ*) are reported in ppm relative to TMS, and signal multiplicities are denoted as *s* (singlet), *d* (doublet), *t* (triplet), and *m* (multiplet). FTIR spectra were recorded to verify functional groups. Liquid samples were analyzed using a Thermo Fisher Nicolet iS5 spectrometer from Waltham, MA, United States with an iD5 ATR diamond crystal, while solid samples were examined on a PerkinElmer Frontier 01, Shelton, CT, USA using the KBr pellet method. Spectra were collected over 400–4100 cm^−1^.

#### 3.3.2. Water Content

The water content of the synthesized PILs and HPILs was determined using a coulometric Karl Fischer auto titrator, Mettler Toledo V30, Columbus, Ohio serial number B440100667. Approximately 0.2 g of each ionic liquid was introduced into the reagent via a volumetric Karl Fischer setup equipped with a Stromboli oven. The analysis was performed at 443.15 K for 15 min to ensure complete moisture evaporation. The water content was expressed as a percentage of the sample weight.

#### 3.3.3. Thermal Stability

The thermal stability of PILs and HPILs was studied using the PerkinElmer STA 6000 thermogravimetric analyser from Shelton, CT, USA in a temperature range from 303.15 K to 973.15 K at a rate of 283.15 K min^−1^ under a nitrogen flow of 20 mL min^−1^. Results were reported in the form of onset temperature, (*T_onse__t_*), and decomposition temperature, (*T_max_*).

#### 3.3.4. Heat Capacity, C_p_

The heat capacity of the PILs and HPILs was analyzed using Differential Scanning Calorimetry with a TA Instruments Q2000 system, New Castle, Delaware within a temperature range of 293.15 to 333.15 K.

#### 3.3.5. Density, ρ

The density of the synthesized HPILs was measured using a 2 mL glass pycnometer for the liquid-state HPILs and a gas pycnometer for the solid-state HPIL. For liquid HPIL, the empty glass pycnometer was weighed, filled completely with the HPIL, and weighed again. The pycnometer containing the sample was placed in a water bath at the required temperature for 15 to 20 min to achieve thermal equilibrium, then reweighed. This procedure was repeated three times for each temperature within the range of 293.15 K to 333.15 K. The average mass of the sample at each temperature was used to determine the density according to Equation (13).

(13) $$\rho_{HPILs}=\frac{\left(m_{final}-m_{initial}\right)}{V_{HPILs}}$$

where $m_{final}$ is the mass of HPILs with glass pycnometer, $m_{initial}$ is the mass of empty glass pycnometer, and $V_{HPILs}$ is the known volume of HPILs (2 mL).

For solid HPIL, its density was measured at 298.15 K using a Micromeritics AccuPyc II TEC gas pycnometer, Micromeritics, Norcross, GA, USA, with helium as the displacement medium. Sample mass was determined by difference, and the instrument software calculated density from the measured volume after ten purge cycles.

#### 3.3.6. Refractive Index Measurement

The refractive index of the HPILs was measured in triplicate using an ATAGO RX-5000 Alpha Digital Refractometer, Tokyo, Japan, at temperatures within the range of 293.15 K to 333.15 K. The validation test was performed using standard organic solvents supplied by the manufacturer to ensure accuracy.

### 3.4. CO_2_ Absorption Measurement

**Figure 14:**
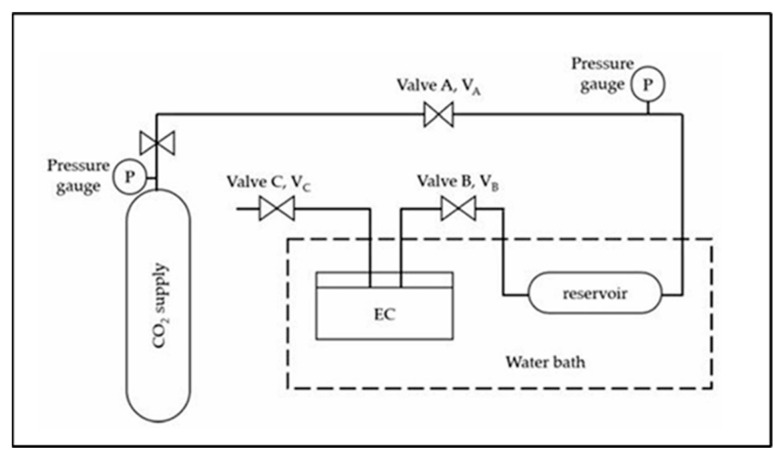
Schematic diagram of the solubility cell used for CO_2_ absorption measurements [7].

The solubility of CO_2_ in the HPILs was determined using the pressure-drop technique with a custom-built solubility cell, as illustrated in Figure 14 and adapted from Rahim et al. [7]. For each run, a pre-weighed amount of HPILs was loaded into a 15 mL stainless steel equilibrium cell and degassed using a vacuum pump. The cell was immersed in a thermostatic water bath maintained at 298.15 K. CO_2_ from the storage vessel was compressed to the desired pressure in the reservoir (V_A_–V_B_) and allowed to stabilize. Once stabilized, CO_2_ was introduced into the equilibrium cell via opening valve V_B_. The system was maintained until equilibrium was reached, which took approximately 120–180 min depending on the ionic liquid. This procedure was repeated at temperatures of 313.15 K and 333.15 K over a pressure range of 1 to 20 bar to evaluate the effect of temperature and pressure on CO_2_ solubility.

The CO_2_ absorption performance of the HPILs was calculated using the following equation:

(14) $$n_{{CO}_{2}}=\;\frac{P_{ini}V_{res}}{Z_{ini}{(P}_{ini}{,T}_{ini})RT_{ini}}-\frac{P_{eq}(V_{total}-\;V_{HPILs})}{Z_{eq}{(P}_{eq},T_{eq})RT_{eq}}$$

where $n_{{CO}_{2}}$ is the number of moles CO_2_ absorbed by HPILs, $P_{ini}{,T}_{ini}$ is the initial pressure and temperature, $P_{eq},T_{eq}$ is the equilibrium pressure and temperature, $V_{res}$ is the volume of CO_2_ in the reservoir, $V_{total}{,V}_{HPILs}$ is the total system volume and liquid adsorbent volume, $Z_{ini},Z_{eq}$ is the compressibility factor at the initial point and equilibrium, calculated using the Peng–Robison equation of state at selected pressure and temperature, and R is the value of the universal gas constant (0.0821 L atm K^−1^ mol^−1^).

The solubility of CO_2_ was expressed as mole fraction calculated using Equation (15):

(15) $$x_{{CO}_{2}}=\frac{n_{{CO}_{2}}}{n_{{CO}_{2}}+n_{HPILs}}$$

where $x_{{CO}_{2}}$ is the mole fraction of CO_2_ absorbed by HPILs, $n_{{CO}_{2}}$ is the number of moles CO_2_ absorbed by HPILs, and $n_{HPILs}$ is the number of moles HPILs used the system. Additionally, Henry’s Law constant was determined by fitting the experimental data to Equation (16):

(16) $$P_{{CO}_{2}}=\;{K_{H}x}_{{CO}_{2}}$$

where $P_{{CO}_{2}}$ is the CO_2_ partial pressure, $x_{{CO}_{2}}$ is the physical absorption value,${\;K}_{H}$ is the Henry’s law constant in HPILs in bar. A similar procedure was also applied to determine the absorption of CH_4_, which was later used for comparison.

The CO_2_ absorption data were used to identify the HPIL with the highest absorption capacity, which was subsequently subjected to a recyclability test. To investigate potential interactions or chemical pathways between CO_2_ and the HPILs, an FTIR analysis was conducted within 20 min after the CO_2_ absorption process.

### 3.5. Recyclability of Hydrophobic Protic Ionic Liquids (HPILs) in CO_2_ Absorption

The recyclability of the HPILs with the highest CO_2_ absorption capacity was evaluated over two consecutive cycles. After each absorption experiment, the CO_2_-loaded HPIL was transferred into an equilibrium cell and regenerated by heating in an oven at 333.15 K for 4 h to remove the absorbed CO_2_. The regenerated HPIL was then reused for subsequent absorption cycles. This procedure was adapted from Rahim et al. and Li et al. [7,18], with minor modifications.

## 4. Conclusions

In conclusion, three HPILs were successfully synthesized, characterized, and evaluated for their CO_2_ absorption performance at temperatures of 298.15, 313.15, and 333.15 K and pressures up to 20 bar. The CO_2_ solubility in all studied HPILs increased with pressure, while a negative correlation with temperature was observed. The alkyl chain length of the cation showed a pronounced effect on CO_2_ absorption capacity, attributed to the increase in free volume (*V_f_*). In addition, the solubility of CH_4_ in these HPILs was significantly lower than that of CO_2_, demonstrating their high selectivity toward CO_2_. Recyclability tests conducted for [BEHA][Tf_2_N] at all temperatures over two consecutive cycles revealed consistent CO_2_ uptake and excellent structural stability, indicating that this ionic liquid is a promising alternative to conventional amine-based solvents such as MEA for CO_2_ capture applications.

## Figures and Tables

**Table 1:** The values of decomposition temperature (*T_onset_*) and peak temperature (*T_max_*) for PILs and HPILs, determined by thermogravimetric analysis.

PILs/HPILs	*T_onset_* (K)	*T_max_* (K)
[DEA][Cl]	478.33	508.26
[TEA][Cl]	480.36	511.00
[BEHA][Cl]	478.18	505.73
[DEA][Tf_2_N]	635.13	677.41
[TEA][Tf_2_N]	672.09	710.75
[BEHA][Tf_2_N]	614.62	651.91

**Table 2:** Density values of HPILs measured at 298.15 K.

HPILs	Density (g·cm^−3^)
[DEA][Tf_2_N]	1.6510
[TEA][Tf_2_N]	1.4377
[BEHA][Tf_2_N]	1.2004

**Table 3:** Fitting parameters to correlate the density of HPILs and calculated *SD*.

HPILs	*D*_1_	*D*_0_	*SD*
[TEA][Tf_2_N]	−0.0008	1.6722	0.0126
[BEHA][Tf_2_N]	−0.0007	1.4082	0.0112

**Table 4:** The values of molar mass, α, *V_m_*, S°, *U_latt_*, *V*, *R_m_*, and *V_f_* for HPILs.

Temperature (K)	Molar Mass (g mol^−1^)	$\alpha \times 10^{-4}$ (K^−1^)	$V_{m}\times 10^{-2}$ (cm^3^ mol^−1^)	S° (J K^−1^ mol^−1^)	*U_latt_* (kJ mol^−1^)	*V* (nm^3^)	*R_m_* (cm^3^ mol^−1^)	*V_f_* (cm^3^ mol^−1^)
[TEA][Tf_2_N]	382.35							
293.15		5.55	2.658	579.64	411.94	0.4413	65.20	200.58
303.15		5.58	2.666	581.37	411.62	0.4427	64.93	201.68
313.15		5.61	2.682	584.69	411.01	0.4454	64.93	203.30
323.15		5.64	2.699	588.18	410.37	0.4482	64.97	205.00
333.15		5.67	2.716	591.68	409.73	0.4510	65.02	206.57
[BEHA][Tf_2_N]	522.62							
293.15		5.80	4.352	930.23	365.25	0.7226	112.44	322.72
303.15		5.83	4.369	933.77	364.90	0.7254	112.10	324.76
313.15		5.86	4.391	938.40	364.46	0.7292	111.91	327.19
323.15		5.90	4.429	946.18	363.72	0.7354	112.12	330.74
333.15		5.93	4.452	950.95	363.27	0.7392	111.98	333.18

**Table 5:** Fitting parameters to correlate *n_d_* of HPILs and calculated *SD*.

HPILs	*R*_1_	*R*_0_	*SD*
[TEA][Tf_2_N]	−0.0003	1.4848	0.0042
[BEHA][Tf_2_N]	−0.0003	1.5247	0.0054

**Table 6:** The *K_H_* values of HPILs at temperatures of 298.15 K, 313.15 K, and 333.15 K.

HPILs	*K_H_* (bar)		
	T = 298.15 K	T = 313.15 K	T = 333.15 K
[DEA][Tf_2_N]	38.70	39.29	44.09
[TEA][Tf_2_N]	30.12	32.81	36.33
[BEHA][Tf_2_N]	25.15	28.28	33.87

**Table 7:** Calculated partial molar enthalpy ($\Delta h_{2}$) and entropy (Δ*s*_2_) of CO_2_ absorption for [BEHA][Tf_2_N], [TEA][Tf_2_N], and [DEA][Tf_2_N]

HPILs	$\mathrm{Enthalpy},\;\Delta h_{2}$ $\left(\frac{kJ}{mol}\right)$	$\mathrm{Entropy},\;\Delta s_{2}$ $\left(\frac{J}{mol\cdot K}\right)$
[DEA][Tf_2_N]	−3.14	−10.02
[TEA][Tf_2_N]	−4.42	−14.03
[BEHA][Tf_2_N]	−7.05	−22.38

**Table 8:** Comparison of Henry’s law constants (*K_H_*) for CO_2_ and CH_4_ absorption in the synthesized 3HPILs at 298.15 K.

HPILs	*K_H_* (bar)	
	CO_2_ (T = 298.15 K)	CH_4_ (T = 298.15 K)
[DEA][Tf_2_N]	38.70	48.05
[TEA][Tf_2_N]	30.12	35.92
[BEHA][Tf_2_N]	25.15	32.33

## Data Availability

The original contributions presented in this study are included in the article/Supplementary Materials.

## Supplementary Materials

The following supporting information can be downloaded at: https://www.mdpi.com/article/10.3390/molecules30244674/s1, Figures S1–S12: ^1^H NMR and ^13^C NMR analyses of all PILs and HPILs. Figures S13 and S14: FTIR Spectra of TEA, BEHA, and their corresponding salts. Table S1: Heat capacity values of all PILs and HPILs measured from 293.15 K to 333.15 K. Table S2: Density values of [TEA][Tf_2_N] and [BEHA][Tf_2_N] measured from 293.15 to 333.15 K. Table S3: Refractive index values of [TEA][Tf_2_N] and [BEHA][Tf_2_N] measured from 293.15 to 333.15 K. Refractive index Figures S15 and S16: Stacked FTIR of [TEA][Tf_2_N] and [DEA][Tf_2_N] before and after CO_2_ absorption. Figures S17 and S18: Stacked FTIR spectra of [BEHA][Tf_2_N] for the fresh sample and after CO_2_ desorption in Cycle 1 and Cycle 2 at 313.15 K and 333.15 K. Figures S19 and S20: Stacked ^1^H NMR spectra of [BEHA][Tf_2_N] for the fresh sample and after CO_2_ desorption in Cycle 1 and Cycle 2 at 313.15 K and 333.15 K.

## Abbreviations

The following abbreviations are used in this manuscript:

*α*	Thermal expansion coefficient
*ρ*	Density
$\Delta h_{2}$	Partial molar enthalpy of the gaseous solute in the liquid phase
$\Delta s_{2}$	Partial molar entropy of the gaseous solute in the liquid phase
[BEHA][Ac]	Bis(2-ethylhexyl)ammonium acetate
[BEHA][BA]	Bis(2-ethylhexyl)ammonium butyrate
[BEHA][Cl]	Bis(2-ethylhexyl)ammonium chloride
[BEHA][Tf_2_N]	Bis(2-ethylhexyl)ammonium bis(trifluoromethylsulfonyl)imide
[BEHA]^+^	Bis(2-ethylhexyl)ammonium
[C_4_Py][Tf_2_N]	1-butylpyridium bis(trifluoromethylsulfonyl)imide
[DEA][Cl]	Diethylammonium chloride
[DEA][Tf_2_N]	Diethylammonium bis(trifluoromethylsulfonyl)imide
[DEA]^+^	Diethylammonium
[HMIM][Tf_2_N]	1-hexyl-3-methylimidazolium bis(trifluoromethylsulfonyl)imide
[TEA][Cl]	Triethylammonium chloride
[TEA][Tf_2_N]	Triethylammonium bis(trifluoromethylsulfonyl)imide
[TEA]^+^	Triethylammonium
ATR	Attenuated total reflectance
BEHA	Bis(2-ethylhexyl)amine
CAS	Chemical abstracts service number
CDCl_3_	Deuterated chloroform
CH_4_	Methane
CO_2_	Carbon dioxide
*C_p_*	Heat capacity
DEA	Diethylamine
DMSO	Dimethyl sulfoxide
DSC	Differential scanning calorimetry
FTIR	Fourier transform infrared spectroscopy
H_2_S	Hydrogen sulphide
HPILs	Hydrophobic protic ionic liquids
ILs	Ionic liquids
KBr	Potassium bromide
*K_H_*	Henry’s law constants
LiTf_2_N	Lithium bis(trifluoromethylsulfonyl)imide
MEA	Monoethanolamine
$n_{{CO}_{2}}$	Number of moles CO_2_ absorbed by ionic liquid
*n_d_*	Refractive index values
$n_{HPILs}$	Number of moles HPILs used in the system
NMR	Nuclear magnetic resonance
*P_eq_*	Equilibrium pressure
$P_{{CO}_{2}}$	CO_2_ partial pressure
PILs	Protic ionic liquids
*P_ini_*	Initial pressure
*R*	Universal gas constant value
RI	Refractive index
*R_m_*	Molar refraction
*S°*	Standard molar entropy
*SD*	Standard deviation
*T*	Absolute temperature
TEA	Triethylamine
*T_eq_*	Equilibrium temperature
Tf_2_N^−^	Bis(trifluoromethylsulfonyl)imide anion
TGA	Thermogravimetric analysis
*T_ini_*	Initial temperature
*T_max_*	Peak temperature
TMS	Tetramethylsilane
*T_onset_*	Onset temperature
TSILs	Task-specific ionic liquids
*U_latt_*	Standard lattice energy
*V*	Molecular volume
*V_f_*	Free volume
*V_HPILs_*	Liquid adsorbent volume
*V_m_*	Molar volume
*V_res_*	Volume of the CO_2_ in the reservoir
*V_total_*	Total system volume
$x_{{CO}_{2}}$	Mole fraction of CO_2_ dissolved in the ionic liquid
$x_{2}$	Mole fraction of the gaseous solute at saturation
*Z_eqb_*	Equilibrium compressibility factor
*Z_ini_*	Initial compressibility factor
