# Supplementary material for: Tuning CO2 Absorption in Hydrophobic Protic Ionic Liquids via Temperature and Structure
Source: Molecules. 2025 Dec 5;30(24):4674. doi: 10.3390/molecules30244674 (PMC12736301; doi:10.3390/molecules30244674)
Supplement: Supplementary file 1 [file molecules-30-04674-s001.zip › molecules-3984342-supplementary.pdf]

SUPPLEMENTARY MATERIALS

CO_2 Uptake in Hydrophobic Protic Ionic Liquids: Influences of Temperature and Structure

1.0 NMR Analysis

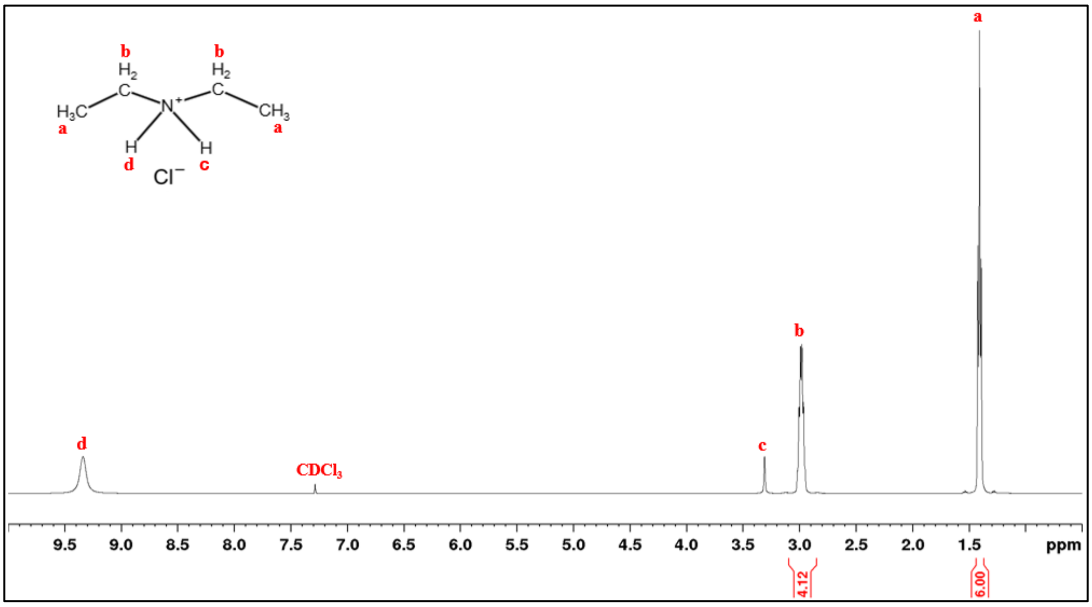

Figure S1: ^{1}H NMR spectrum of [DEA][Cl] recorded in $CDCl_3$.

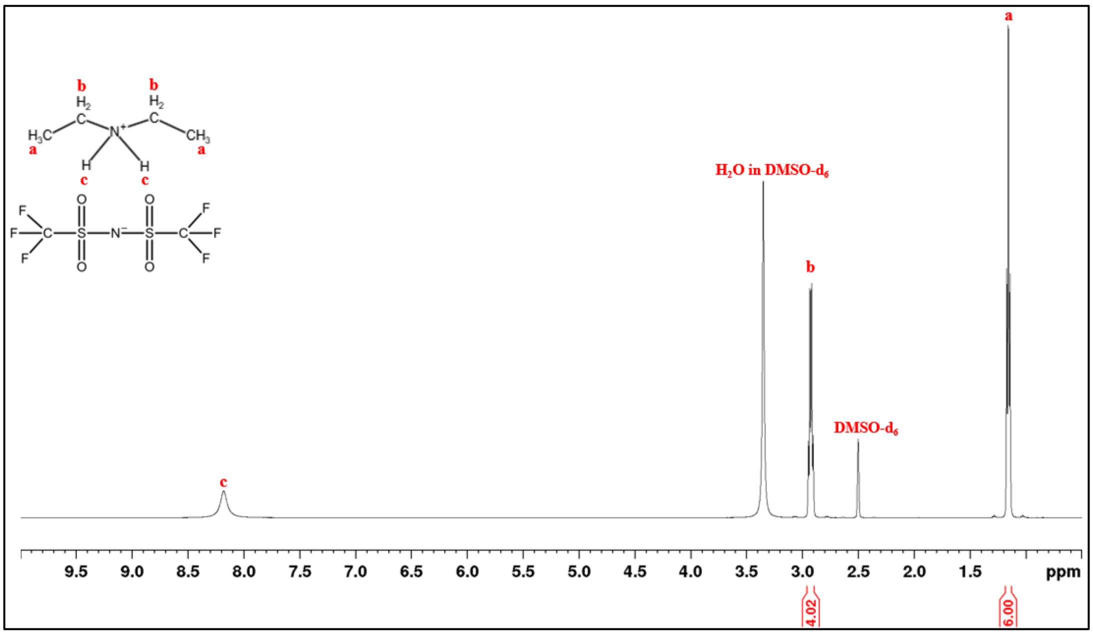

Figure S2: ^{1}H NMR spectrum of [DEA][Tf_2N] recorded in DMSO-d_6.

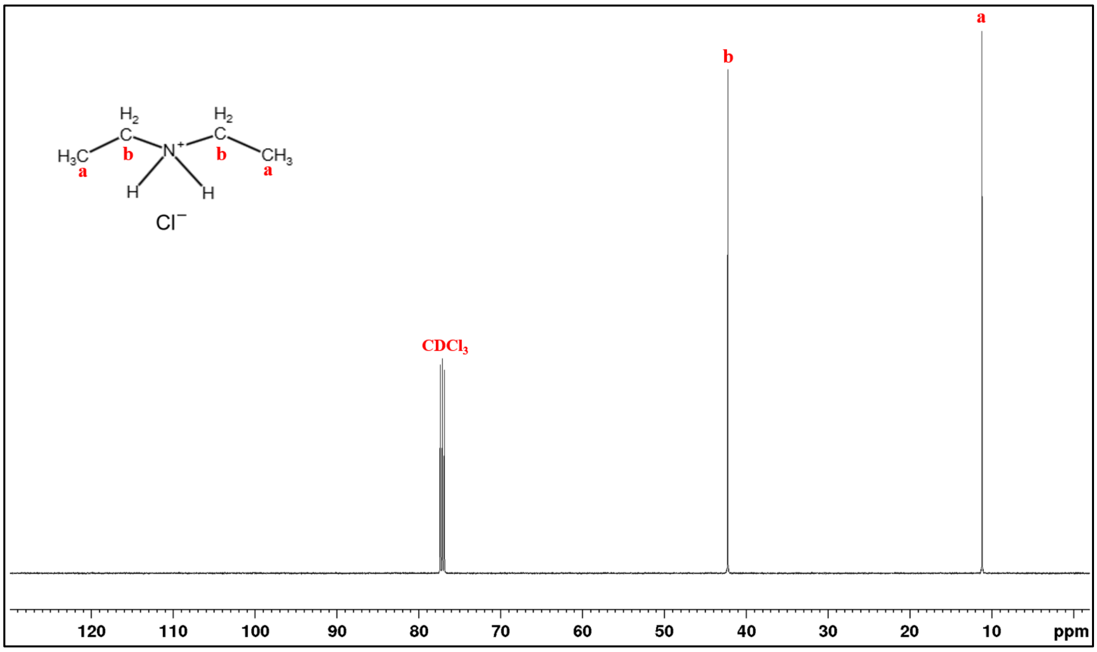

Figure S3: ^{13}C NMR spectrum of [DEA][Cl] recorded in CDCl3.

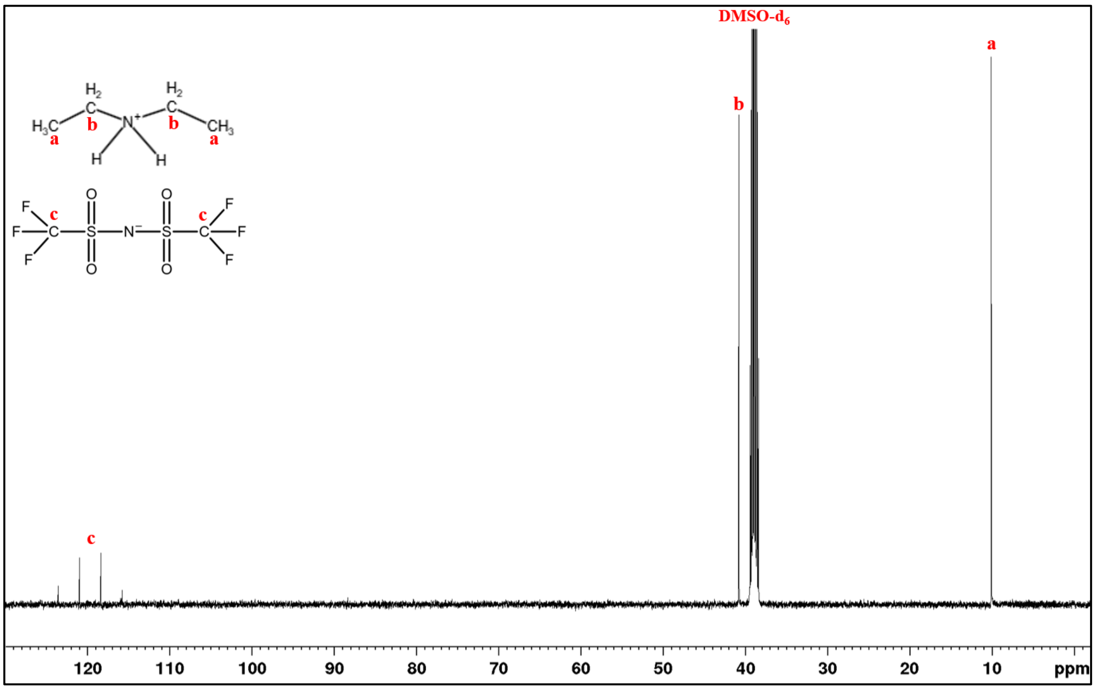

Figure S4: ^{13}C NMR spectrum of [DEA][Tf2N] recorded in DMSO-d6.

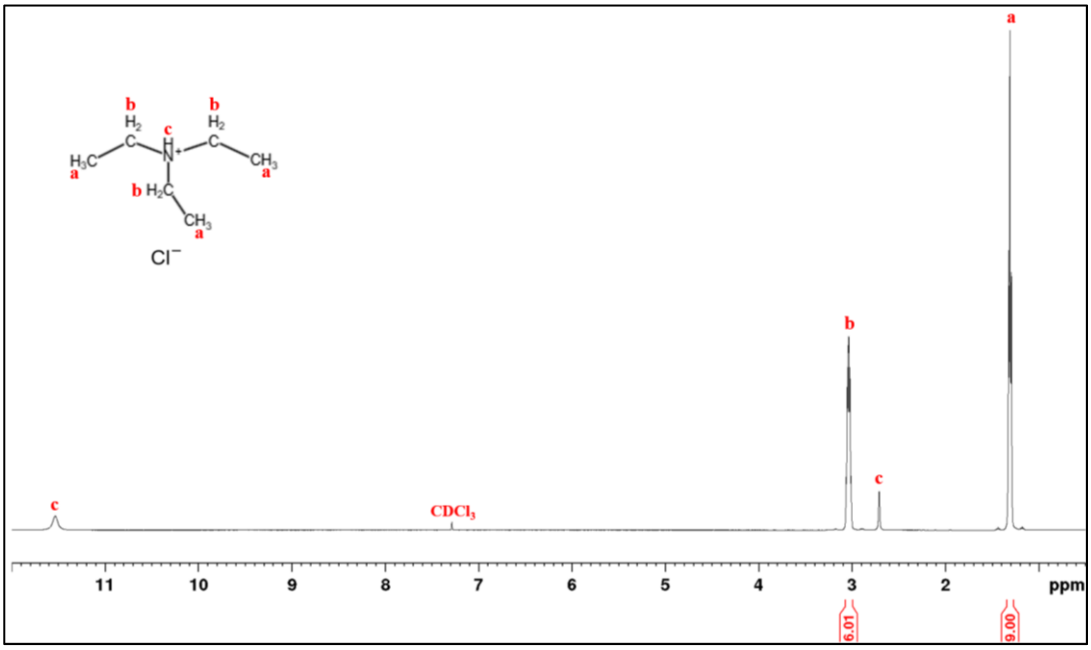

Figure S5: ^{1}H NMR spectrum of [TEA][Cl] recorded in $CDCl_3$.

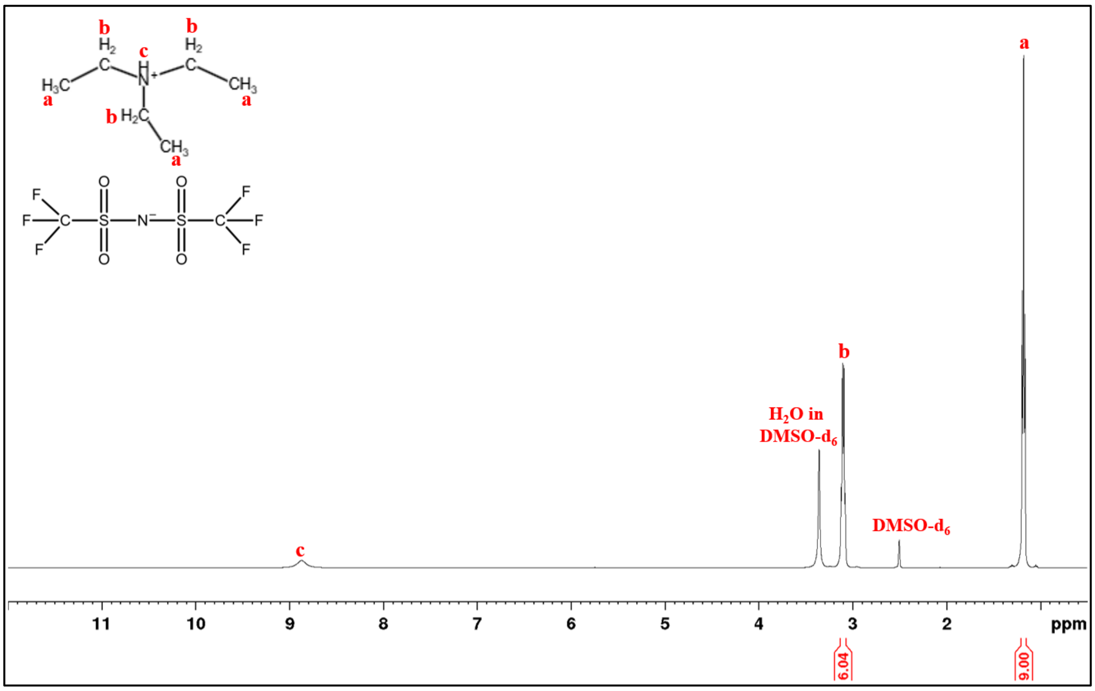

Figure S6: ^{1}H NMR spectrum of [TEA][Tf_2N] recorded in DMSO-d_6.

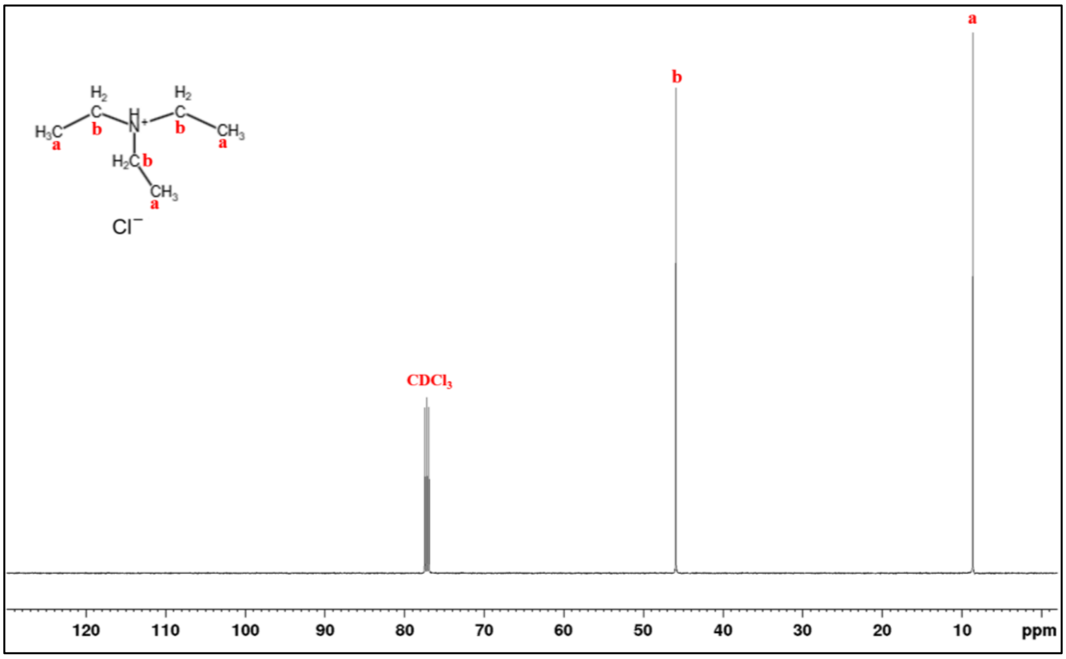

Figure S7: ^{13}C NMR spectrum of [TEA][Cl] recorded in $CDCl_3$.

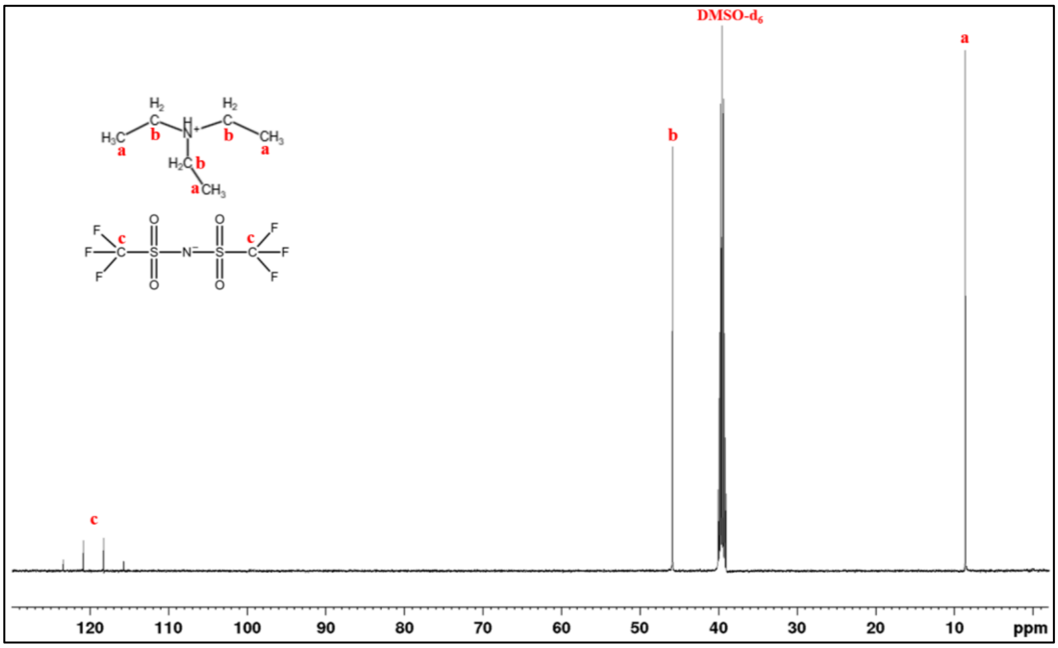

Figure S8: ^{13}C NMR spectrum of [TEA][Tf_2N] recorded in DMSO-d_6.

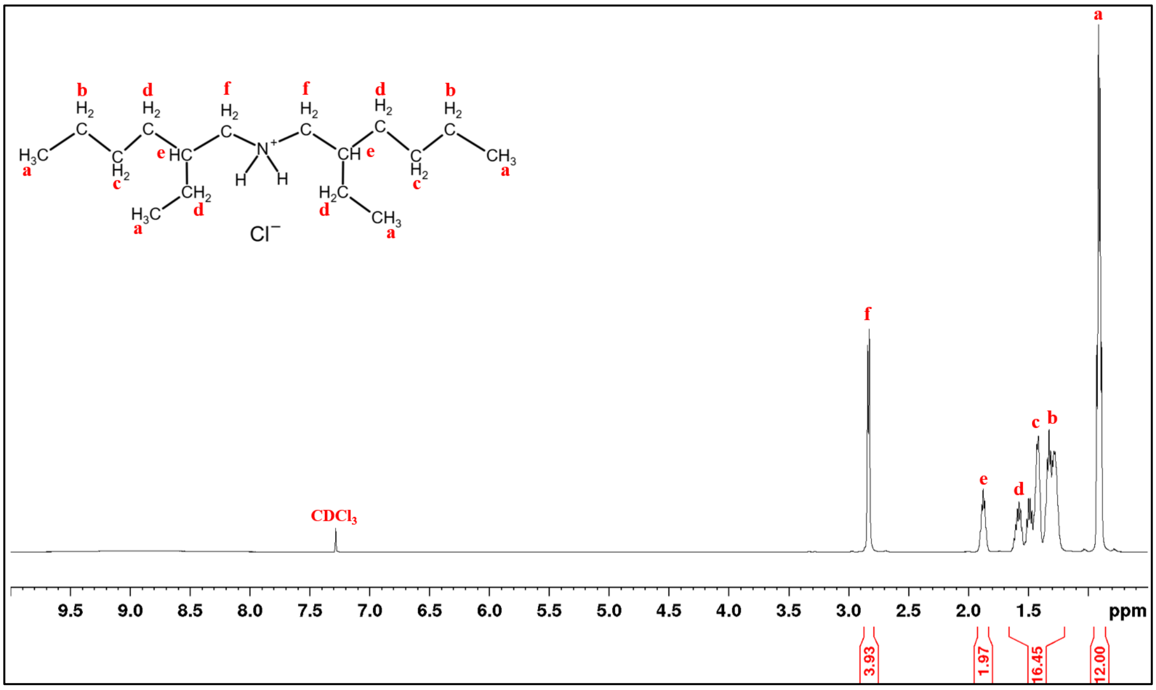

Figure S9: ^{1}H NMR spectrum of [BEHA][Cl] recorded in CDCl3.

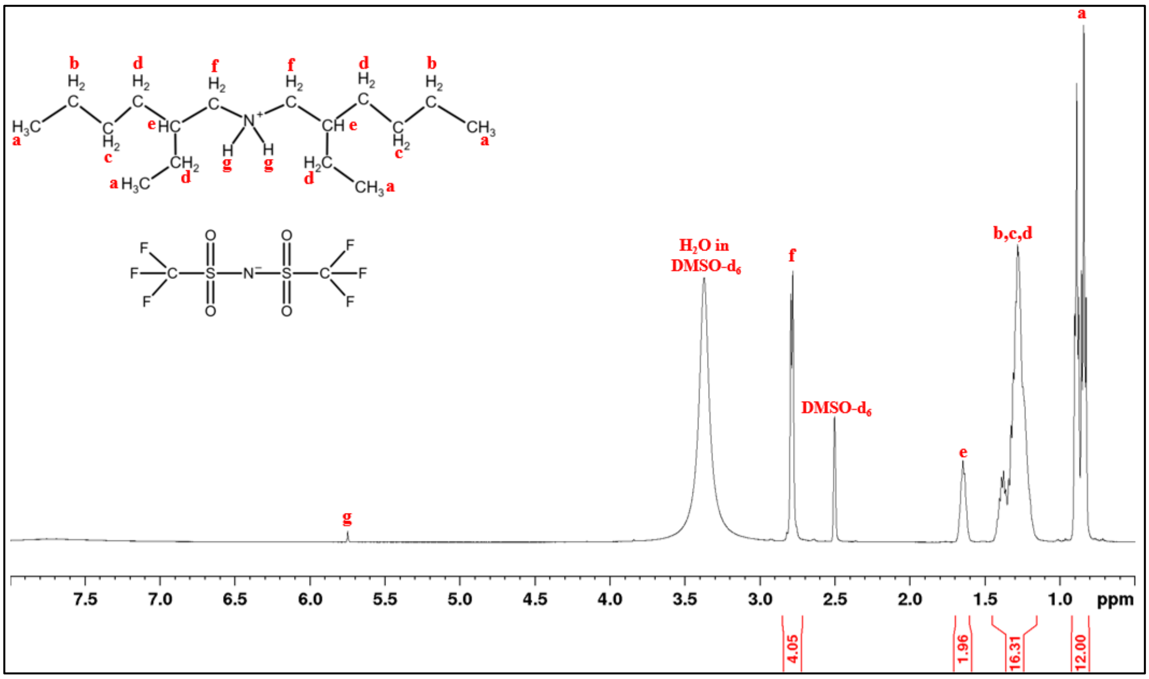

Figure S10: ^{1}H NMR spectrum of [BEHA][Tf2N] recorded in DMSO-d6.

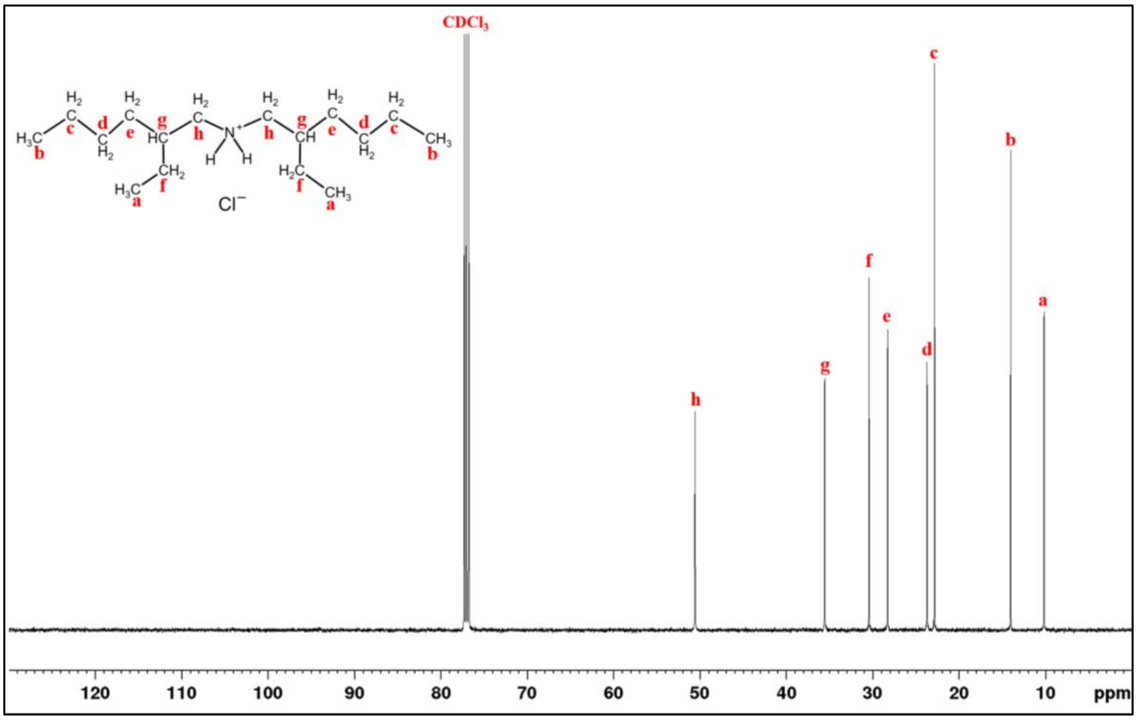

Figure S11: ^{13}C NMR spectrum of [BEHA][Cl] recorded in $CDCl_3$.

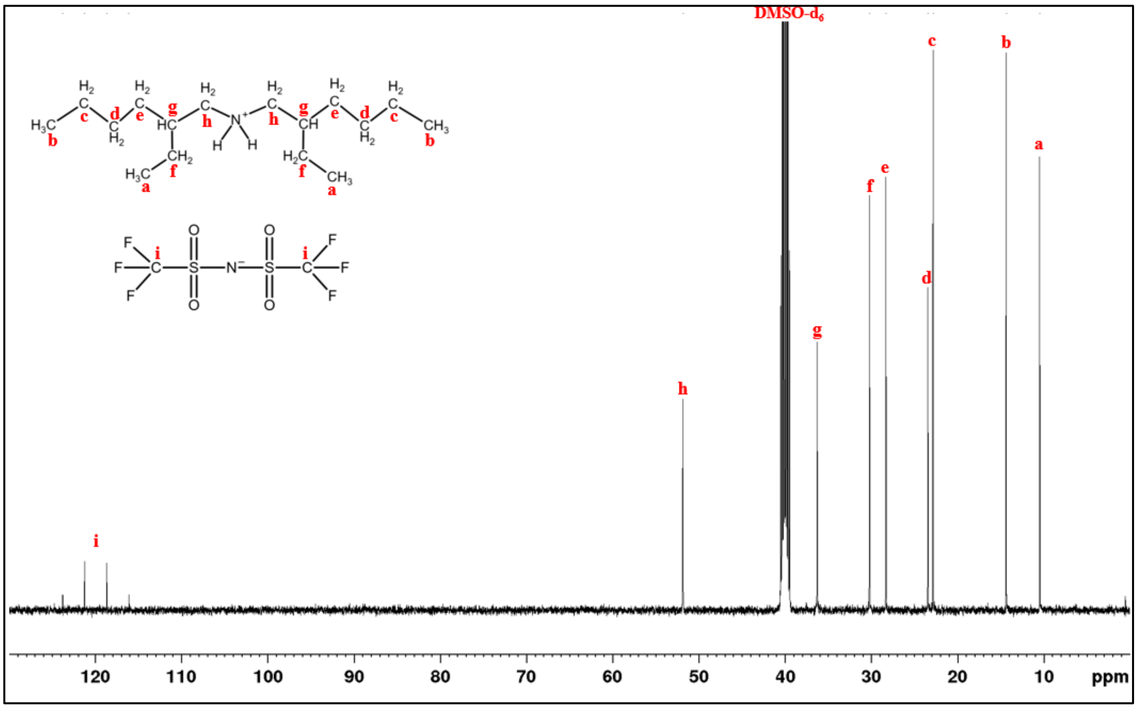

Figure S12: ^{13}C NMR spectrum of [BEHA][Tf_2N] recorded in DMSO-d_6.

Summary of NMR Chemical Shift Data and Water Content for Synthesized Protic Ionic Liquids

The summarized ^{1}H and ^{13}C NMR data for each ionic liquid, together with their measured water contents, are presented below for reference:

[DEA][Cl]: ^{1}H NMR (500 MHz, $CDCl_3$): δ 9.339 [s, 1H, RN^+H_2], 3.309 [s, 1H, R-NH], 2.979 [q, 4H, N-CH_2], 1.408 [t, 6H, CH_3]. ^{13}C NMR (125 MHz, $CDCl_3$): δ 42.31, 11.17. Water content: 1.579%.

[DEA][Tf_2N]: ^{1}H NMR (500 MHz, DMSO): δ 8.179 [s, 1H, RN^+H_2], 2.935 [q, 4H, N-CH_2], 1.160 [t, 6H, CH_3]. ^{13}C NMR (125 MHz, DMSO): δ 123.49, 120.93, 118.37, 115.81, 41.54, 11.15. Water content: 0.142%.

[TEA][Cl]: ^{1}H NMR (500 MHz, $CDCl_3$): δ 11.535 [s, 1H, RN^+H], 3.035 [q, 6H, N-CH_2], 2.710 [s, 1H, RN^+H], 1.310 [t, 9H, CH_3]. ^{13}C NMR (125 MHz, $CDCl_3$): δ 45.92, 8.63. Water content: 3.319%.

[TEA][Tf_2N]: ^{1}H NMR (500 MHz, DMSO): δ 8.874 [s, 1H, RN^+H], 3.095 [q, 6H, N-CH_2], 1.179 [t, 9H, CH_3]. ^{13}C NMR (125 MHz, DMSO): δ 123.50, 120.94, 118.38, 115.82, 45.98, 8.74. Water content: 0.476%.

[BEHA][Cl]: ^{1}H NMR (500 MHz, $CDCl_3$): δ 2.826 [d, 4H, N-CH_2], 1.874 [m, 2H, N-CH_2-CH], 1.413 [m, 16H, aliphatic CH_2], 0.912 [t, 12H, -CH_3]. ^{13}C NMR (125 MHz, $CDCl_3$): δ 50.59, 35.57, 30.43, 28.27, 23.71, 22.85, 14.02, 10.17. Water content: 0.735%.

[BEHA][Tf_2N]: ^{1}H NMR (500 MHz, DMSO): δ 7.63 [s, 1H, RN^+H_2], δ 2.796 [d, 4H, N-CH_2], 1.639 [m, 2H, N-CH_2-CH], 1.286 [m, 16H, aliphatic CH_2], 0.844 [m, 12H, -CH_3]. ^{13}C NMR (125 MHz, DMSO): δ 123.81, 121.25, 118.69, 116.13, 51.86, 36.28, 30.21, 28.31, 23.45, 22.85, 14.35, 10.47. Water content: 0.376%.

2.0 FTIR Analysis

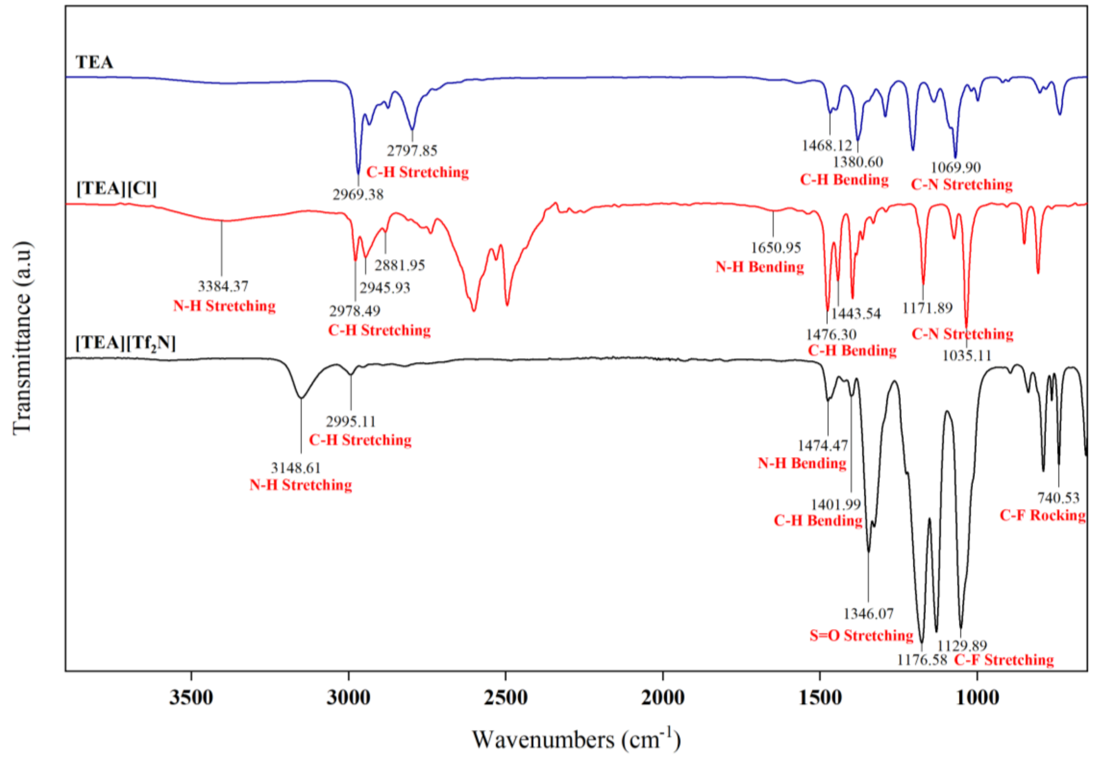

Figure S13: Stacked FTIR spectra of TEA, [TEA][Cl], and [TEA][Tf2N].

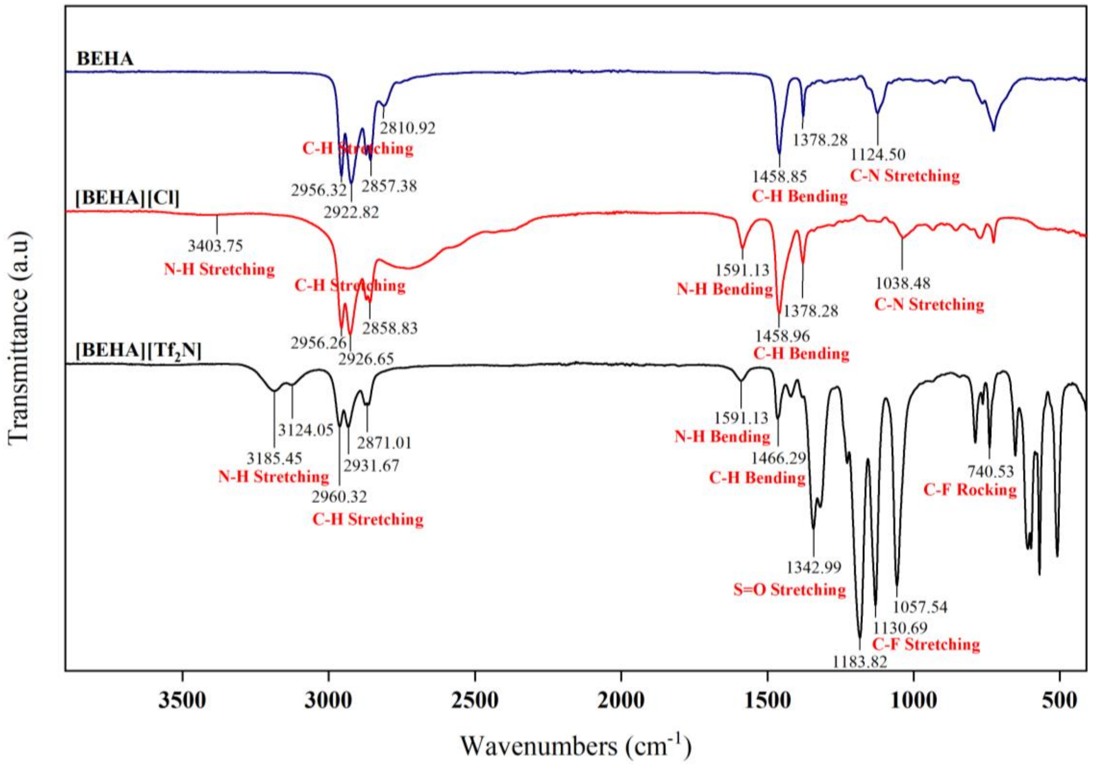

Figure S14: Stacked FTIR spectra of BEHA, [BEHA][Cl] and [BEHA][Tf2N].

3.0 Heat Capacity Values

Table S1: Heat Capacity, C_p values of all PILs at 293.15K - 333.15K.

Temperature (K)	Heat Capacity, C_p (J/ g K)					
	[DEA][Tf_2N]	[TEA][Tf_2N]	[BEHA][Tf_2N]	[DEA][Cl]	[TEA][Cl]	[BEHA][Cl]
293.15	2.503	2.352	1.394	3.322	2.802	2.443
298.15	2.653	2.403	1.455	3.422	2.902	2.484
303.15	2.715	2.455	1.514	3.535	3.015	2.525
308.15	2.772	2.509	1.571	3.622	3.135	2.566
313.15	2.829	2.566	1.630	3.665	3.248	2.608
318.15	2.926	2.622	1.691	3.736	3.347	2.630
323.15	2.963	2.682	1.752	3.803	3.476	2.668
328.15	3.155	2.742	1.814	3.899	3.600	2.718
333.15	3.318	2.804	1.878	4.002	3.734	2.773

4.0 Density Values

Table S2: Density values of [TEA][Tf_2N] and [BEHA][Tf_2N] measured at 293.15K - 333.15K.

Temperature (K)	Density ($g\ cm^{-3}$)	
	[TEA][Tf_2N]	[BEHA][Tf_2N]
293.15	1.4386	1.2010
303.15	1.4341	1.1963
313.15	1.4255	1.1902
323.15	1.4166	1.1801
333.15	1.4078	1.1740

5.0 Refractive Index Values

Table S3: Refractive index values of [TEA][Tf_2N] and [BEHA][Tf_2N] measured at 293.15K - 333.15K.

Temperature (K)	Refractive Index	
	[TEA][Tf_2N]	[BEHA][Tf_2N]
293.15	1.4054	1.4301
303.15	1.4021	1.4267
313.15	1.3993	1.4234
323.15	1.3968	1.4202
333.15	1.3944	1.4172

6.0 FTIR Analysis of HPILs after CO_2 Absorption

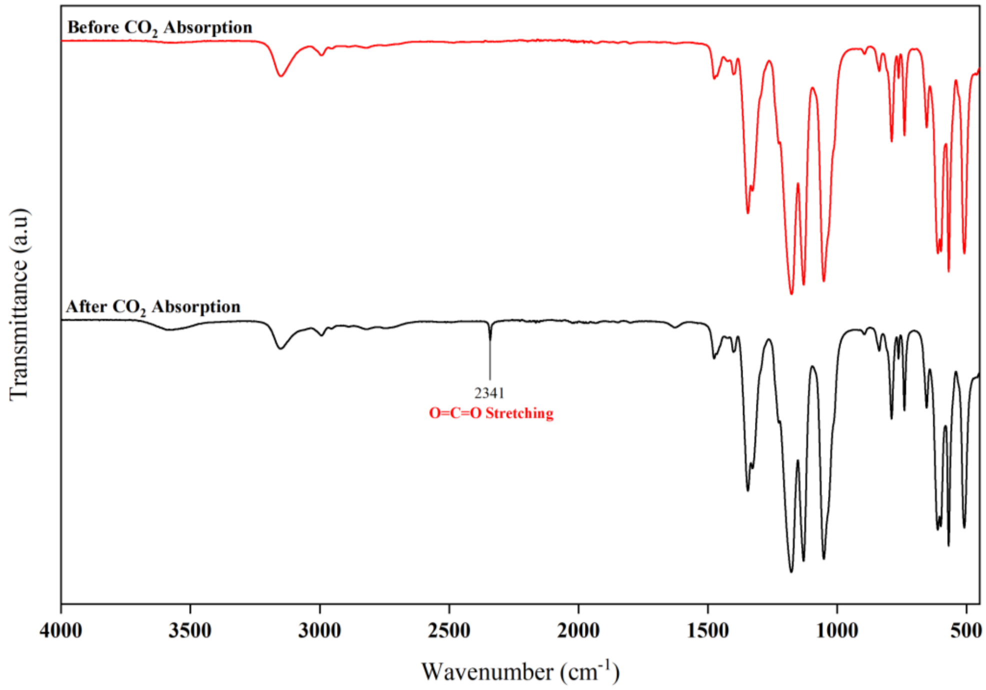

Figure S15: Stacked FTIR spectra of [TEA][Tf$_2$N] before and after CO_2 absorption.

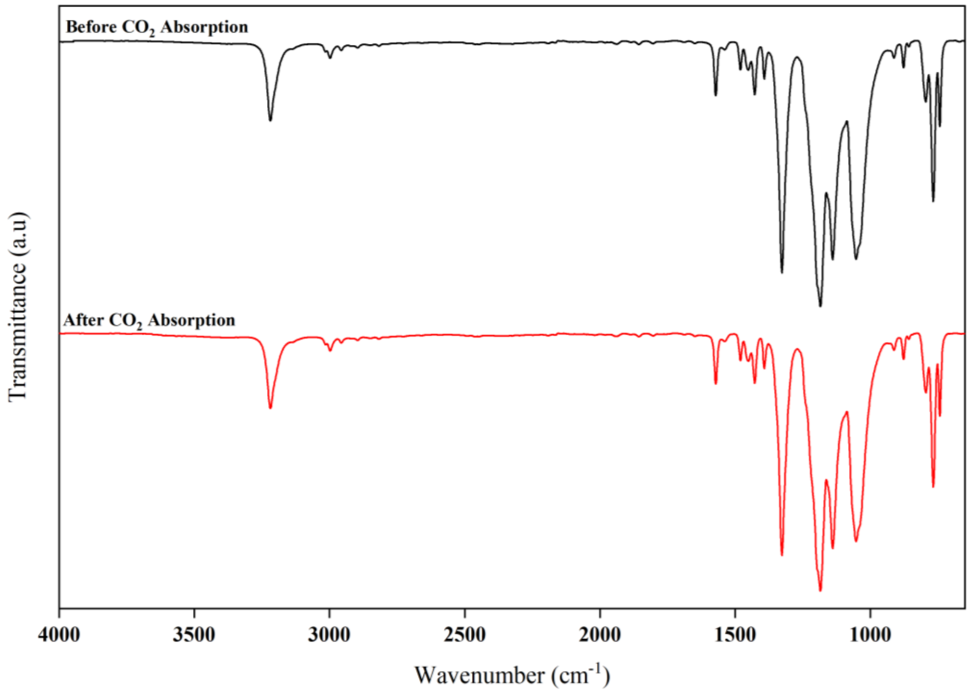

Figure S16: Stacked FTIR spectra of [DEA][Tf$_2$N] before and after CO_2 absorption.

7.0 FTIR and ^{1}H NMR Analyses of HPILs after CO_2 Desorption

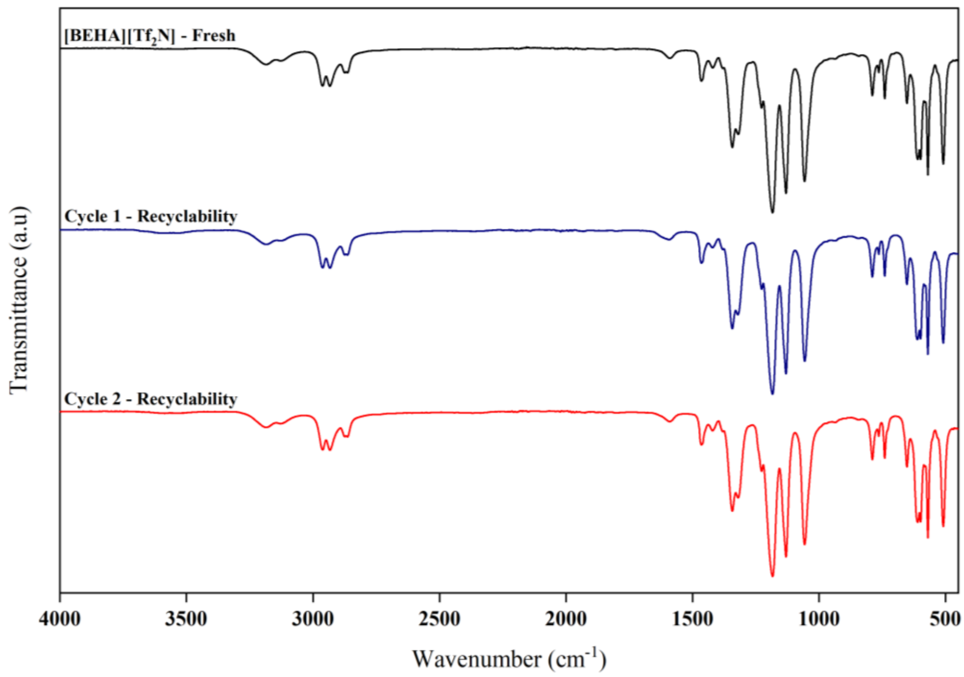

Figure S17: Stacked FTIR spectra of [BEHA][Tf2N] for the fresh sample and after CO_2 desorption in Cycle 1 and Cycle 2 at 313.15 K.

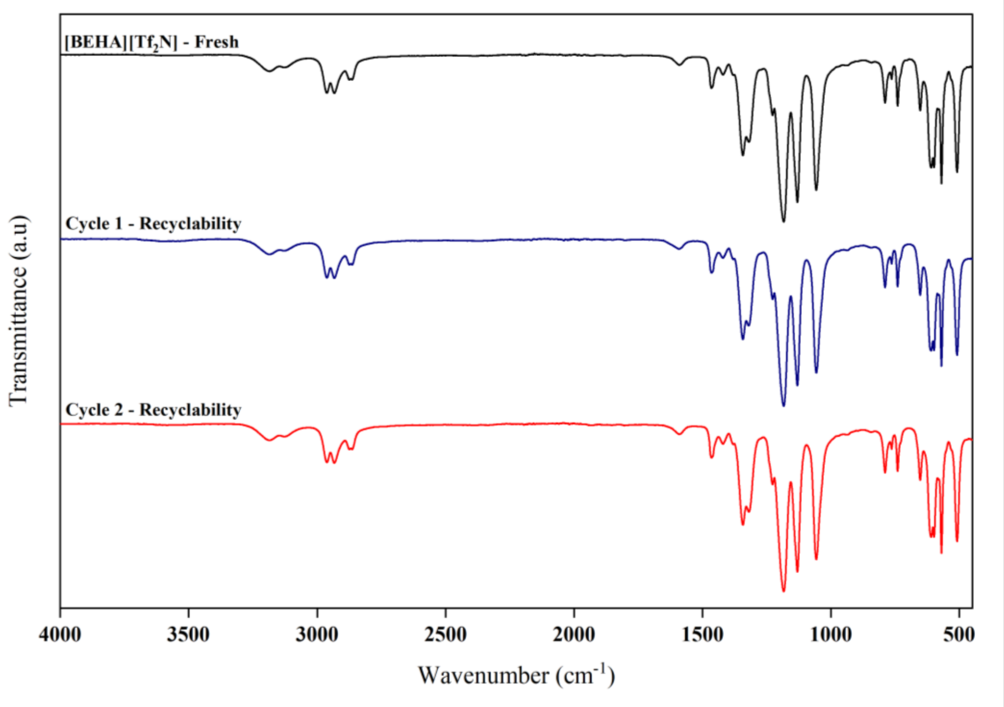

Figure S18: Stacked FTIR spectra of [BEHA][Tf2N] for the fresh sample and after CO_2 desorption in Cycle 1 and Cycle 2 at 333.15 K.

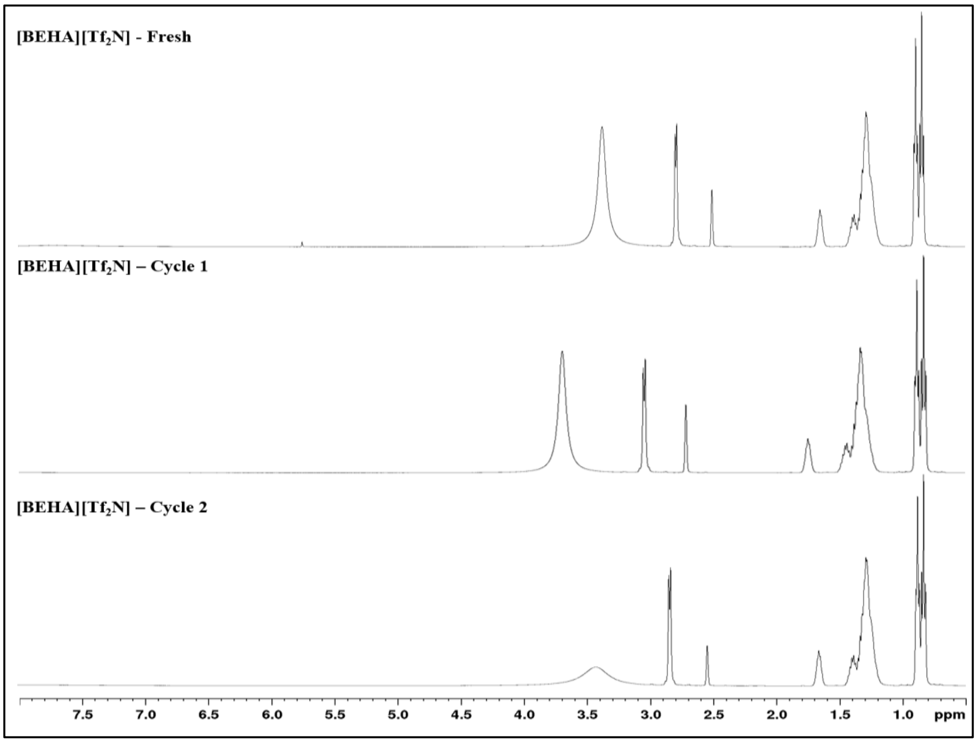

Figure S19: Stacked ^{1}H NMR spectra of [BEHA][Tf2N] for the fresh sample and after CO_2 desorption in Cycle 1 and Cycle 2 at 313.15 K.

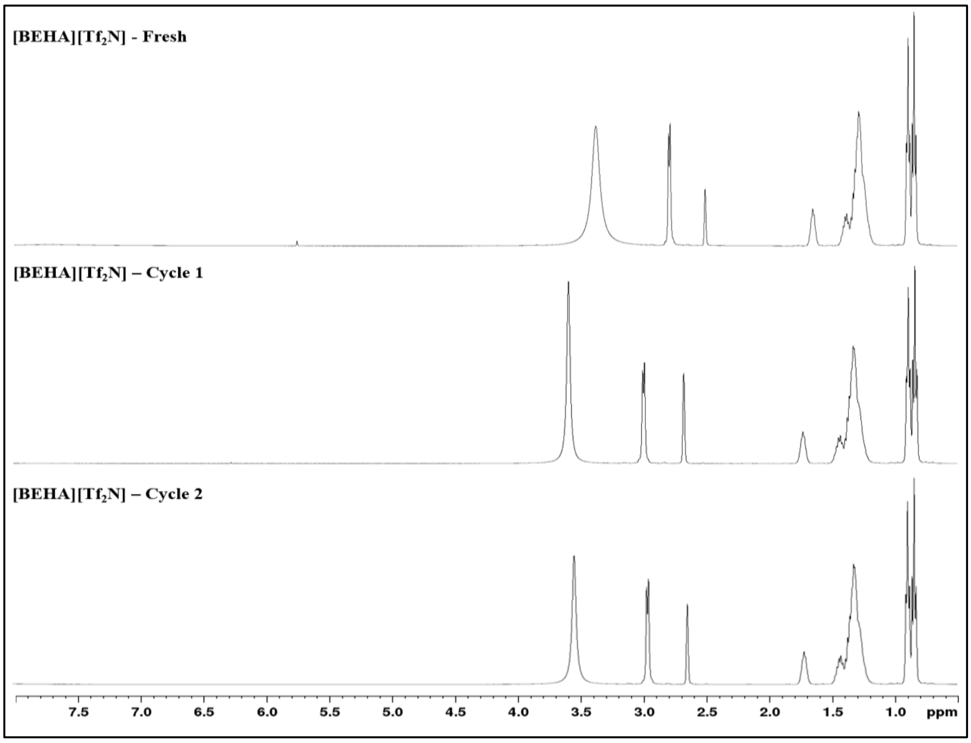

Figure S20: Stacked ^{1}H NMR spectra of [BEHA][Tf2N] for the fresh sample and after CO_2 desorption in Cycle 1 and Cycle 2 at 333.15 K.
